# Taxa-area relationship of aquatic fungi on deciduous leaves

**DOI:** 10.1371/journal.pone.0181545

**Published:** 2017-07-18

**Authors:** Sofia Duarte, Fernanda Cássio, Cláudia Pascoal, Felix Bärlocher

**Affiliations:** 1 Centre of Molecular and Environmental Biology (CBMA), Department of Biology, University of Minho, Braga, Portugal; 2 Institute of Science and Innovation for Bio-Sustainability (IB-S), University of Minho, Braga, Portugal; 3 Department of Biology, Mount Allison University, Sackville, New Brunswick, Canada; Leibniz Institut - Deutsche Sammlung von Mikroorganismen und Zellkulturen GmbH, GERMANY

## Abstract

One of the fundamental patterns in macroecology is the increase in the number of observed taxa with size of sampled area. For microbes, the shape of this relationship remains less clear. The current study assessed the diversity of aquatic fungi, by the traditional approach based on conidial morphology (captures reproducing aquatic hyphomycetes) and next generation sequencing (NGS; captures other fungi as well), on graded sizes of alder leaves (0.6 to 13.6 cm^2^). Leaves were submerged in two streams in geographically distant locations: the Oliveira Stream in Portugal and the Boss Brook in Canada. Decay rates of alder leaves and fungal sporulation rates did not differ between streams. Fungal biomass was higher in Boss Brook than in Oliveira Stream, and in both streams almost 100% of the reads belonged to active fungal taxa. In general, larger leaf areas tended to harbour more fungi, but these findings were not consistent between techniques. Morphospecies-based diversity increased with leaf area in Boss Brook, but not in Oliveira Stream; metabarcoding data showed an opposite trend. The higher resolution of metabarcoding resulted in steeper taxa-accumulation curves than morphospecies-based assessments (fungal conidia morphology). Fungal communities assessed by metabarcoding were spatially structured by leaf area in both streams. Metabarcoding promises greater resolution to assess biodiversity patterns in aquatic fungi and may be more accurate for assessing taxa-area relationships and local to global diversity ratios.

## Introduction

The observed number of taxa almost always increases with sampled area [1, 2], a relationship that has been widely studied for animals and plants [3, 4]. On the other hand, the traditional view concerning microbes has long been that “everything is everywhere, but the environment selects” [5]. This hypothesis claims that small organisms (< 2 mm), commonly referred to as microorganisms, have a high potential of dispersal and therefore present cosmopolitan distributions, with little or no evidence of historical or geographical constraints [5–7]. In the case of larger organisms, such as animals and plants, it is assumed that if taxa or species are not randomly distributed, larger areas would contain more species differing in their niche requirements [3, 4]. Alternatively or in addition, a positive taxa/species-area relationship will appear simply due to greater sampling effort when larger areas are examined [4]. With microbes, increasing the area of a survey would only marginally increase species richness of a sample [7]. For instance, Fenchel and Finlay [6] found as many protist morphospecies in a single lake as previously described from the entire world. However, advances in DNA-based identification have revolutionized how microbial diversity is assessed, by allowing discriminating among morphologically indistinguishable species. This development has raised doubts about the ubiquity of microbes [8, 9]. Indeed, Taylor et al. [10] argued that for fungi, the inferred geographic range of a species depends upon the methods of species recognition. Species defined by morphology may show global geographic ranges, but genetic isolation, followed by reproductive isolation, often precede a recognizable morphological change.

The most appropriate mathematical function describing the relationship of the species/taxa-area has been debated over the years [4]. The three most widely used descriptions of the taxa-area relationships (TAR) have been the exponential, the power and the logistic curve [4]; in addition, rectangular hyperbolae have occasionally been used (e.g. [11]). Most commonly, the relationship is described by a power law:

$$S=c\;*A^{z}$$ (1)

where *S* corresponds to the number of taxa or species, *c* is a constant that is empirically derived from the taxon or species and the specific location, *A* is the sampled area and *z* the rate at which taxa or species increase with area [1, 2, 9], but the shape of the curve will influence *z* values [4]. For various groups of macroorganisms, z values vary between 0.1 and 0.3 in contiguous habitats, and between 0.3 and 0.4 in spatially separated habitats with little or no exchange (discrete islands) [7, 12]. On the other hand, earlier studies with microbes in contiguous habitats suggest that z values decline to much lower values between 0.002 and 0.07 [13–17]. In discrete islands the slope may be indistinguishable from that observed for macroorganisms—ca. 0.3 in the study by Bell et al. [7]. No quantitative explanation has been yet described for the enormous range found for z values for microorganisms. But the disparity between sample size and community size, that largely exceeds that of macroorganisms, can partly explain the differences and is clearly related to the use of molecular methods to assess microbial diversity. For instance, most molecular methods analyse small samples harbouring numerous and species-rich microbial communities [13]. It has been also suggested that significant microbial taxa-area relationships will only be observed when changes in community structure within samples correlate with area [13].

Aquatic hyphomycetes provide a tractable microbial system to study species-area relationships. These are an ecologically defined group of fungi that play key roles in plant-litter decomposition in running waters [11, 18]. Fungal extracellular enzymes initiate the conversion of recalcitrant organic carbon into fungal biomass (mycelium and conidia), which increases litter quality for invertebrate detritivores, and macerates the leaf matrix, resulting in the release of fine particulate organic matter [11, 18]. Within a few days after colonizing a substrate, many fungal species produce vast amounts of conidia that enter the water column and are transported downstream [19]. These conidia are typically tetraradiate, variously branched or scolecoid (worm-like) [20]. Their characteristic shapes often allow identification to species and insights into fungal community structure [20].

The wide geographic distribution of aquatic hyphomycetes [21] suggests excellent dispersal abilities. Aquatic hyphomycetes are also reported to occur in different substrates that include wood, twigs and leaves from different tree species, and therefore most species are believed to have broad fundamental niches [12, 22], which would tend to flatten the relationship between taxa/species richness and sampled area [12]. However, the geographic distance between streams can also influence community composition, with closer streams sharing similar species, suggesting some spatial turnover in stream-dwelling decomposer fungi [23, 24]. On the other hand, in a recent literature review aquatic hyphomycetes seemed to be more influenced by environmental factors found at local to regional scales (e.g. stream water nitrate, conductivity) than by the geographic distance between streams [24]. In addition, molecular studies have reported different genotypes in different leaf species, suggesting a degree of intraspecific substrate specialization and, thus, some niche differentiation [25]. However, the mechanisms that influence their actual occurrence at any given location, and the dissimilarity between fundamental and realised niches, are not well understood [12].

Nevertheless, at a much smaller scale within streams, the number of aquatic hyphomycete morphospecies was significantly correlated with the initial size of the sampled substrate (wood blocks [26], oak and maple leaves [27, 28], and larch and spruce needles [27]). These studies were based on the identification of morphospecies, which may overlook cryptic species, thus blurring global-scale biogeographical patterns and taxa-area relationships inferences.

The current study seeks to clarify the species-area relationship of aquatic fungi on graded sizes of alder leaves submerged in two streams with similar physical and chemical characteristics but located at two different geographic locations. One stream is in the northwest of Portugal (Oliveira Stream) and the other on the east coast of Canada (Boss Brook). Diversity of fungi was assessed by conidium morphology (morphospecies, which captures primarily actively reproducing aquatic hyphomycetes). The other approach, metabarcoding of DNA and RNA extracted from submerged leaf disks, may also capture other aquatic fungi, or even transient terrestrial fungi. For the purposes of this study, all fungi documented by either approach were considered “aquatic”. The source of DNA used for sequencing and OTU identification may be living or dead hyphae and spores, or extracellular and environmental debris [29]. RNA is much less likely to occur and be stable outside a metabolically active cell; any OTU based on cDNA is therefore likely to originate from an active taxon [30].

We hypothesized that: i) with both morphospecies- and molecular-based approaches, the number of fungal taxa will increase with the leaf area at both geographic locations; ii) the slope of the taxa or species-area curves will be steeper when using metabarcoding since its higher resolution is expected to reveal more unique and potentially endemic species; iii) there will be a greater number of DNA than RNA based OTUs; and iv) RNA based OTUs will be a subset of DNA based OTUs.

## Materials and methods

### Field experiment

Two streams were selected at two temperate but geographically distant locations. The first stream, Oliveira Stream, is a 4^th^ order stream located in the Northwest of Portugal (41°35.107’N, 8°13.305’W, altitude: 232 m). The riparian vegetation is dominated by *Alnus glutinosa* Gaertn., *Quercus robur* L. and *Castanea sativa* Miller, the stream bed consists of rocks and pebbles [31], and at the sampling site the stream is 2 to 3 m wide and 15 to 45 cm deep [32]. Previous experiments indicated pH near neutrality (6.9) and low values of conductivity (37 μS cm^-1^) and nutrients in the stream water (20.0 μg PO_4_^3-^ L^-1^ and 518 μg N-NO_3_^-^ L^-1^) [31–33]. The second stream, Boss Brook, is a 1^st^ order stream in Fenwick, Nova Scotia (45°43.000’N, 064°09.567’W, altitude: 78 m). The riparian vegetation is dominated by *Betula papyrifera* Marsh, *Acer rubrum* L., *A*. *saccharum* Marsh., *A*. *spicatum* L am. and *Picea glauca* (Moench) Voss; the stream bed is dominated by stones and gravel and at the sampling site the stream is 2 to 3 m wide and 20 to 50 cm deep [34–36]. Previous experiments indicated a slightly acid pH (6.1) and low levels of conductivity (57 μS cm^-1^) and nutrients in the stream water (33.8 μg PO_4_^3-^ L^-1^ and 81.3 μg N-NO_3_^-^ L^-1^) [35]. During the experimental period, water temperature, measured hourly with submerged data loggers (Hobo Pendant UA-001-08, Onset Computer Corp), averaged 12.1°C in Oliveira Stream and 11.4°C in Boss Brook. Temperature was significantly higher in Oliveira Stream (unpaired t-test, P<0.0001).

Air dried alder leaves, collected in autumn 2014 before abscission in the Northwest of Portugal, were leached for 2 h and cut into leaf disks with a 1.2 cm cork borer. Sets of 60 leaf disks were enclosed in fine-mesh bags (0.5 mm mesh size, 20 x 20 cm, 5 replicates) and dried at room temperature. The fine-mesh bags to be immersed in Boss Brook, in Canada, were carefully conditioned in a protected box, in order to minimize any losses during transportation, and were sent to Canada by express air mail. Immersion periods were from 24th March to 13th April 2015, in Oliveira Stream, and 13th May to 8th June 2015, in Boss Brook. After recovery from the stream, leaf disks were immediately separated into sets representing 6 areas (4 replicates per area), and stored in 2-mL Eppendorf tubes containing 1 mL of RNAlater (Sigma-Aldrich). These sets were subsequently used for RNA and DNA analyses. The following quantities (areas) were used: ½ disk (0.6 cm^2^), 1 leaf disk (1.1 cm^2^), 2 leaf disks (2.3 cm^2^), 4 leaf disks (4.5 cm^2^), 8 leaf disks (9.0 cm^2^) and 12 leaf disks (13.6 cm^2^).

The remaining leaves from each bag were transported to the laboratory in a cooling box. In the laboratory, 2 sets of 8 and 24 leaf disks were freeze-dried to estimate ergosterol content and remaining dry mass (± 0.0001 g, Sartorius TE214S), respectively. Additional sets of leaf disks, representing the same six areas used for molecular analyses, were used to induce fungal sporulation.

No specific permissions were required for these locations since they were not located in any national park or other protected area of land or in any private land. In addition, the field studies did not involve endangered or protected species.

### Fungal sporulation

Fungal sporulation was induced by placing leaf disks in 250 mL Erlenmeyer flasks with 75 mL of autoclaved stream water. Flasks were put on an orbital shaker (120 rpm) for 48 h, at 15°C. Sporulation was stopped by the addition of 1 mL of 37% formaldehyde (v/v) (Sigma-Aldrich) and conidial suspensions were mixed with 100 μL Tween 80 (Sigma-Aldrich) at 15% (v/v) in 50 mL Falcon tubes. Appropriate volumes of each replicate were filtered (5 μm pore size, Millipore), and stained with 0.05% (w/v) cotton blue in lactic acid (Fluka). At least 300 conidia per filter were counted and identified under a light microscope to determine the contribution of each species to total conidial production. Fungal sporulation rates were calculated for each species as the number of conidia released per g of leaf dry mass per day.

### Fungal biomass

Fungal biomass was estimated from ergosterol concentration in leaf disks according to Gessner [37]. Lipids were extracted from sets of 8 freeze-dried leaf disks by heating (80°C for 30 min) in 8 g L^-1^ KOH in methanol. The ergosterol was purified by solid phase extraction, eluted in isopropanol and quantified by high performance liquid chromatography (Dionex UltiMate 3000, Thermo Fisher Scientific), using a LiChrospher RP18 column (250 × 4 mm, Merck KGaA). The system was run isocratically with HPLC-grade methanol at 1.4 mL min^-1^ and 33°C. Ergosterol was detected at 282 nm and quantified based on a standard curve of ergosterol in isopropanol (Sigma-Aldrich).

### DNA and RNA processing

Before extractions, RNAlater was poured off from each Eppendorf tube and RNA and DNA extracted from leaf disks using the RNA/DNA purification kit from Norgen (Norgen Biotek Corp.), following the manufacturer’s instructions. RNA and DNA concentrations were measured spectrophotometrically in a NanoDrop ND-1000 (VWR). For each treatment, separate RNA and DNA extracts from 4 replicates were mixed in equal concentrations and used to perform PCR (in the case of RNA, after cDNA synthesis).

cDNA was synthesized from 1 μL of each RNA pool (ca. 10–20 ng) using 0.6 μM of the primers ITS1-F and ITS4 [38] and the OneStep RT-PCR kit (Qiagen) by using 2 μL of OneStep RT-PCR enzyme mix, 1x OneStep RT-PCR buffer, 1x of Q-solution and 0.4 mM of each dNTP, according to the manufacturer’s instructions. The following program was used: i) RT-PCR at 50°C for 30 min, ii) activation of HotStar Taq DNA polymerase and inactivation of reverse transcriptase at 95°C for 15 min, iii) 30 cycles of denaturation at 94°C for 45 sec, annealing at 55°C for 45 sec and extension at 72°C for 1.5 min, and iv) final extension at 72°C for 10 min.

A control for all DNA and RNA pools was performed by using 1x GoTaq Green Master Mix (Promega), 0.4 μM of the primers ITS1-F and ITS4 and 1 μL of DNA (5–10 ng) or RNA extract (10–20 ng) in a final volume of 25 μL. The following program was used: i) initial denaturation of 95°C for 5 min, ii) 30 cycles of denaturation at 95°C for 45 sec, annealing at 55°C for 45 sec and extension at 72°C for 1.5 min, and iii) final extension at 72°C for 10 min. A negative control (nucleases-free water instead of RNA or DNA extracts) was included in all reactions. No amplicons were found in negative controls or PCR of RNA pools, which indicates that there was no DNA contamination in RNA extracts (S1 Fig).

### Illumina MiSeq analysis

Two PCR reactions were conducted for each sample to prepare amplicon libraries for Illumina sequencing. All PCRs were carried out in 25 μL reaction volumes containing 1.5 mM MgCl_2_, 0.2 μM of each primer (ITS1-F and 58A2R), 0.2 mM of each dNTP, 5% DMSO, and 0.02 U μL^-1^ HotStarTaq polymerase (Qiagen). An initial denaturation and enzyme activation step at 96°C for 15 min was followed by amplification for 35 cycles at the following conditions: 96°C for 30 sec, 52°C for 30 sec, and 72°C for 1 min. A final 10 min extension at 72°C completed the protocol in a Mastercycler pro S thermocycler (Eppendorf). The second PCR was conducted with the primers holding a CS1 and CS2 tail (underlined) for adding the barcodes and Illumina adapters to each fragment ((ITS1F-CS1F: ACACTGACGACATGGTTCTACACTTGGTCATTTAGAGGAAGTAA and 58A2R-CS2R: TACGGTAGCAGAGACTTGGTCTCTGCGTTCTTCATCGAT) (5’-3’)). After the second PCR, each amplicon was run in an agarose gel, its concentration measured with picogreen and the DNA normalized to achieve identical concentrations before pooling all amplicons. The pooled library of the desired size was cleaned with AMPure XP beads (Beckman Coulter), and the DNA quality was assessed and quantified with picogreen and LabChip GX (Perkin Elmer, Inc.) and qPCR was used to get a more precise estimation of the concentration of the target fragments. Fungal ITS1 regions from ribosomal rRNA gene and rRNA were then sequenced on an Illumina MiSeq platform, using an Illumina MiSeq PE250 kit, at McGill University and Génome Québec Innovation Centre (http://gqinnovationcenter.com/index.aspx). ITS1 regions were targeted by using ITS1-F and 58AR primers. ITS1-F primer has been reported to discriminate well fungal against plant DNA [39]. Although in previous studies we have targeted ITS2 region in NGS [31, 40], we got some contamination with non-fungal DNA [31], and in this study we wanted to avoid that.

### Sequencing processing and analyses

Raw reads were first scanned for contaminants (e.g. Illumina adapter sequences) and PhiX reads. All unpaired reads were discarded and remaining reads were trimmed to a fixed length (165 bp) before read pair assembly to reconstitute the original amplicons using FLASH software (http://ccb.jhu.edu/software/FLASH/). The trimmed assembled/single-end reads were filtered for quality. This included discarding reads that: i) had an average quality score lower than 30 or ii) more than 10 unidentified Ns and 10 nucleotides below quality 20. Filtered reads were clustered with the Innovation Centre clustering algorithm. Briefly, reads were clustered at 100% identity and clustered/denoised at 99% identity using dnaclust (http://dnaclust.sourceforge.net/). Clusters with an abundance below 3 were discarded. Remaining clusters were scanned for chimeras with UCHIME denovo and UCHIME reference [41] and clustered at 97% (dnaclust) to form the final clusters/OTUs. The taxonomic distribution of the OTUs was performed using the RDP classifier with a modified Greengenes training set built from a concatenation of the Greengenes database, Silva eukaryotes 18S r108, chloroplasts and mitochondria rRNA SSU sequences, using Qiime (http://qiime.org/) [42] software suite. ITS1 database consist of the UNITE ITS database (ITS1-ITS2) regions. The RDP classifier gives a score (0.00 to 1.00) to each taxonomic depth of each OTU. Each taxonomic depth having a score > = 0.5 is kept to reconstruct the final lineage. From these classified OTUs, diversity metrics were obtained by aligning OTU sequences on a Greengenes core reference alignment [43] using the PyNAST aligner [42]. Alignments were filtered to keep only the hypervariable region part of the alignment. Alpha diversity metrics (observed OTUs, Chao-1 richness index and Shannon diversity index) were then computed using Qiime [42]. Further refinements to assess OTUs taxonomic assignment identity, in particular the OTUs with hits with fungal sequences, was performed by blasting individually each OTU against GenBank/nt@ncbi (http://www.ncbi.nlm.nih.gov/genbank/). The final filtered OTU table, with the matrix containing each OTU with hits with fungal sequences present per sample, was then used to assess beta diversity metrics (Bray-Curtis index) and construct the Principal coordinates analysis (PCo) diagram (see next sub-section).

Raw sequences were deposited in the Sequence Read Archives (SRA) of the NCBI under the accession number SRR5116472.

### Statistical analyses

Kolmogorov-Smirnov tests for normality and Levene’s tests for homogeneity of variances were applied to data of temperature, leaf dry mass remaining, ergosterol, sporulation rates and the number of reads [44]. Unpaired t-tests were used to assess differences in temperature, leaf dry mass remaining, ergosterol concentrations and sporulation rates between Oliveira Stream and Boss Brook. Two-way analyses of variance followed by Tukey’s post-hoc tests were used to evaluate differences in the number of reads between total and active communities and between streams.

Michaelis-Menten models were used to establish the relationships between alpha diversity measures (observed OTUs, Chao-1 richness index and Shannon diversity index) and the sampling effort (sequencing depth) as:

V=(Vmax * sequencing depth)/(Km + sequencing depth) (2)

where *V*_*max*_ is the maximum function (observed OTUs, Chao-1 richness index or Shannon diversity index), *K*_*m*_ is the sequencing depth needed to reach half of the maximum function, and the sequencing depth represents the sampling effort. For each site, differences in *V*_*max*_ among leaf areas were assessed using extra sum-of-squares F-test [45].

Principal coordinates analysis (PCo), based on the Bray-Curtis index, was used to ordinate fungal communities based on morphospecies and OTUs with hits for fungi by the two streams (Oliveira Stream versus Boss Brook), leaf area (0.6 to 13.6 cm^2^) and the type of community (total versus active, for metabarcoding assessed diversity). Data were square root transformed prior to construction of similarity matrices. A two-way PERMANOVA was used to assess if stream and leaf area affected fungal morphospecies community structure, while a three-way PERMANOVA, which also included total versus active community was used to assess if these factors affected community structure for metabarcoding derived diversity [46].

The three most widely used models—power, exponential and logistic—discussed in Scheiner [4] were used to establish the relationship between leaf area (LA) and the number of fungal taxa (taxa-area curve, TAR) or the cumulative number of fungal taxa (taxa-accumulation curve, TAC) based on morphospecies or on OTUs with hits for active fungi. The specific equations applied to the data were:

ln(S)=z * ln(A) + c, for the power curve (3)

S=z * ln(A) + c, for the exponential curve (4)

$$S=b/(c\;+\;A^{-z}),\text{ for the logistic curve}$$ (5)

where *S* is the number of fungal taxa or the cumulative number of fungal taxa, based on morphospecies or OTUs with hits for active fungi, *A* is the leaf area, *b* and *c* are constants, and *z* the rate by which the number of taxa increases with leaf area (TAR) or the rate of taxa accumulation (TAC).

Normality and homogeneity of variances analyses, t-tests, ANOVAs and regressions analyses were done in GraphPad Prism v6 (GraphPad software Inc, San Diego, CA, USA) and PCo analyses and PERMANOVAs in Primer v6, both for Windows (Plymouth, UK).

## Results

### Functional parameters

**Fig 1:**
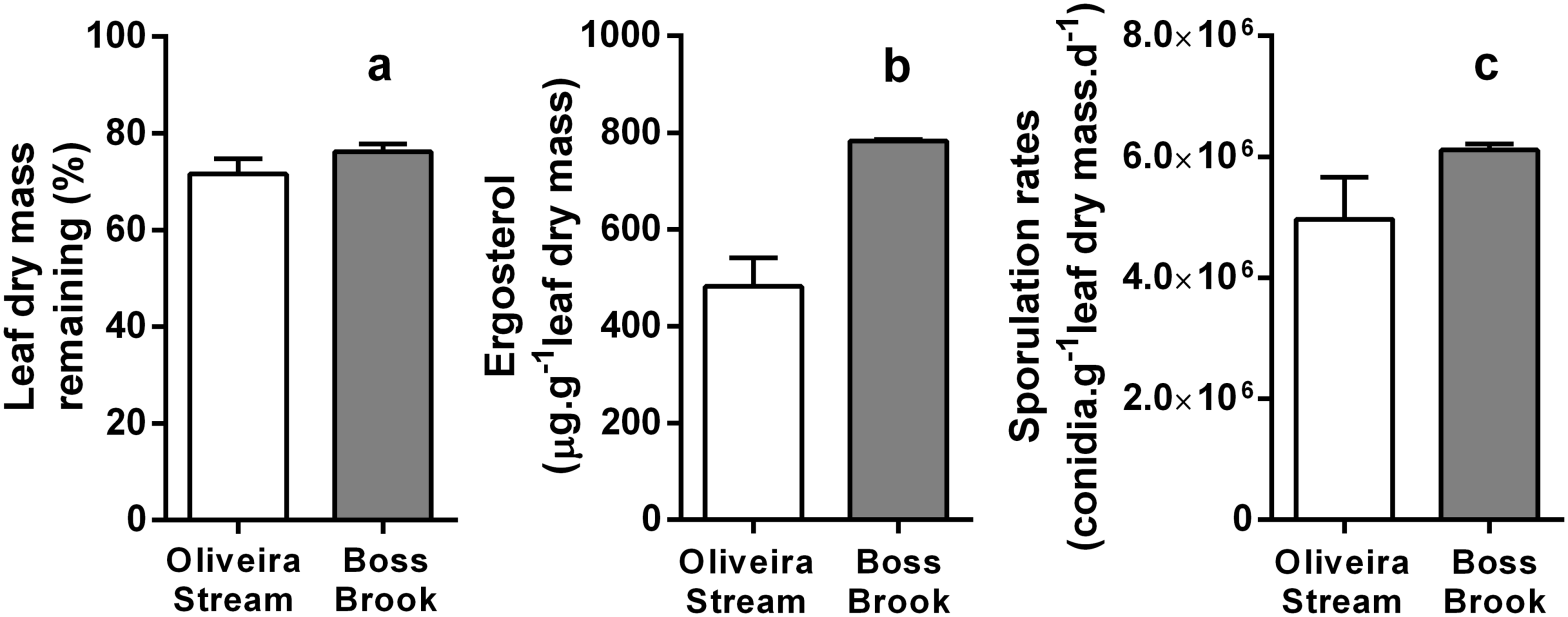
Functional parameters, after 3 weeks of exposure in Oliveira Stream and Boss Brook. Leaf dry mass remaining (percentage of initial values) (a), fungal biomass (as ergosterol concentrations) (b) and sporulation rates (number of conidia per g leaf dry mass per day) (c), Mean + SEM, n = 4.

Leaf dry mass remaining varied between 71.6% and 76.2% for Oliveira Stream and Boss Brook, respectively, and did not significantly differ between streams (unpaired t-test, P = 0.24) (Fig 1a). Ergosterol concentrations in leaf disks were ca. 2 times higher in Boss Brook than in Oliveira Stream (783.2 versus 482.2 μg ergosterol g^-1^ leaf dry mass, respectively; unpaired t-test, P = 0.002) (Fig 1b). Maximum sporulation rates did not significantly differ between streams (5.0 x 10^6^ versus 6.1 x 10^6^ n° conidia g^-1^ leaf dry mass d^-1^, for Oliveira Stream and Boss Brook, respectively; unpaired t-test, P = 0.23) (Fig 1c).

### Fungal diversity: Morphospecies versus OTUs

A total of 47 fungal taxa (morphospecies) were found sporulating from alder leaf disks at Oliveira Stream and Boss Brook (S1 Table). *Clavatospora longibrachiata*, *Dimorphospora foliicola*, *Flagellospora curvula*, *Aquanectria penicillioides* and *Tricladium chaetocladium* contributed the most to total sporulation in Oliveira Stream (maximum between 13.5% and 39.9%), while in Boss Brook *Anguillospora filiformis*, *Articulospora tetracladia* and *F*. *curvula* dominated sporulation (maximum between 36.1% to 46.2%) (S1 Table).

ITS1 amplicon sequencing generated a total of 30,498,302 raw reads, of which 5,391,475 passed quality control and yielded a total of 589 OTUs.

The number of total and active reads did not differ significantly in either stream (2-way ANOVA, P = 0.51) (S2 Fig). Active reads were used for constructing the rarefaction curves (observed OTUs, Chao-1 richness index and Shannon diversity index versus sampling effort—sequencing depth) and for conducting taxa-area and taxa-area accumulation analyses using OTUs with fungal hits; for community analyses, total (DNA) and active (RNA) reads with fungal hits were evaluated separately.

**Fig 2:**
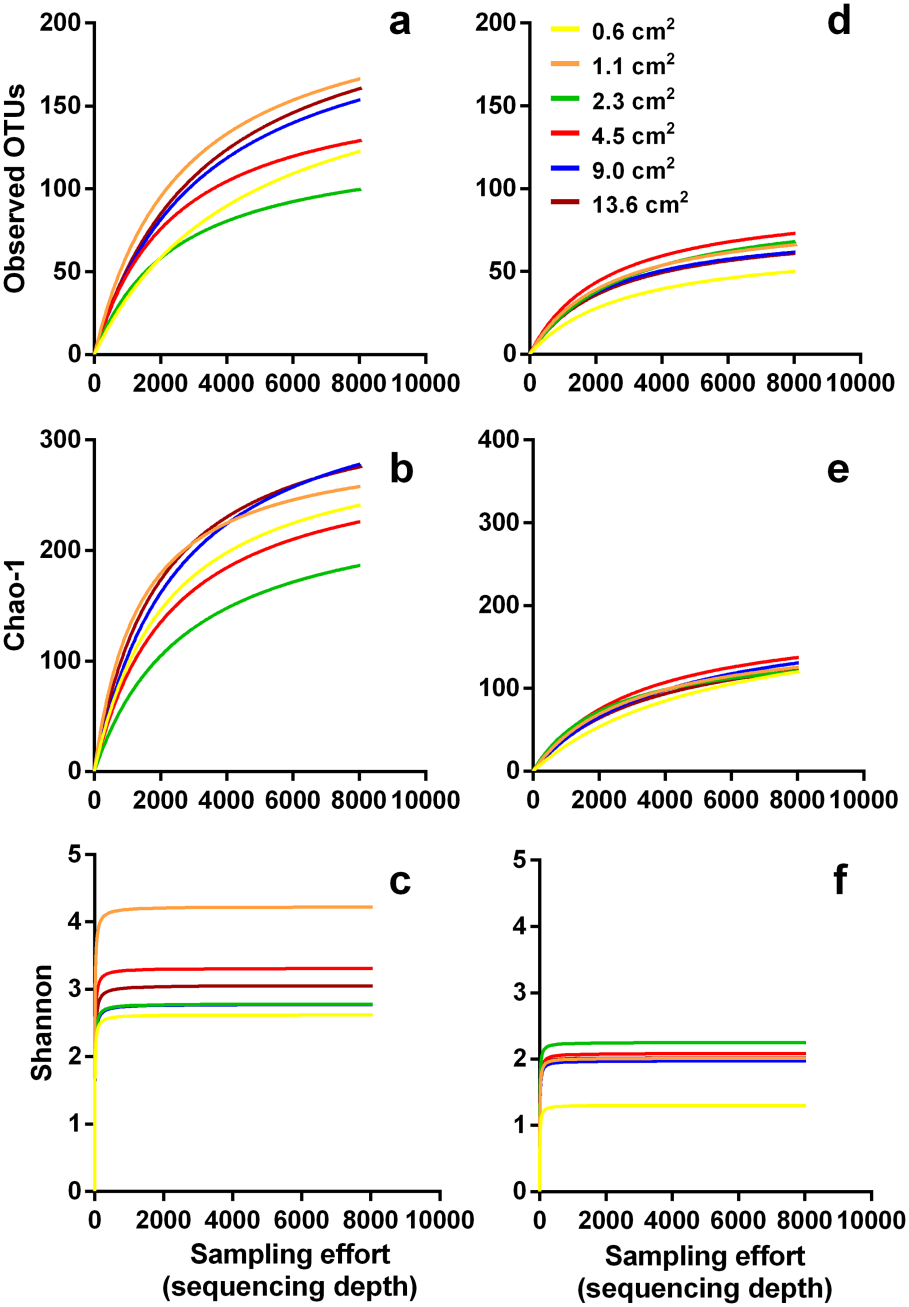
Rarefaction curves on each sampled leaf area and for active taxa. Rarefaction curves were constructed by using observed OTUs, Chao-1 richness index and Shannon diversity index, as a function of the sampling effort (sequencing depth), in Oliveira Stream (a, b, c) and Boss Brook (d, e, f), respectively.

Alpha diversity (observed OTUs, Chao-1 richness index and Shannon diversity index), assessed as a function of the number of reads (Fig 2), seems to be influenced by leaf area. Michaelis-Menten models (Eq 2) gave the best fits for the relationships between alpha diversity and the sampling effort (r^2^ = 0.87 to 0.98, S2 Table), with maximum richness (Vmax) differing significantly among the curves based on different leaf areas (sum-of-squares F-test, P<0.0001, for all comparisons). Maximum values estimated from Michaelis-Menten models for observed OTUs were attained for leaf areas ≥4.5 cm^2^ (228.1 versus 94.51, for Oliveira Stream and Boss Brook, respectively), while maximum Chao-1 richness and Shannon diversity index were attained for lower leaf areas (≥0.6 cm^2^ for Chao-1, 365.8 versus 203.5 and ≥1.1 cm^2^ for Shannon, 4.228 versus 2.249, for Oliveira Stream and Boss Brook, respectively), suggesting that the number of co-existing species quickly approaches a plateau (S2 Table).

Of the 589 OTUs, a total of 368 had close hits for fungal sequences in GenBank. Of the fungal OTUs, 40.5% had close matches with aquatic hyphomycete sequences (S3 Table). Although the number of total and active reads did not differ between streams, unexpectedly the number of total OTUs with fungal hits was lower than the number of active OTUs with fungal hits (109 versus 232, for Oliveira Stream, and 120 versus 219, for Boss Brook) (S3 Table). In both streams, OTUs with hits for *Articulospora tetracladia* EU998923 sequences dominated the number of total and active reads with fungal hits (maximum between 72.5% and 84.3% and 57.0% and 78.5%, for total and active reads, respectively) (S3 Table). Among total reads, other major contributors included OTUs with close hits to Helotiales sp. JN225950 (45.7%), *Flagellospora saccata* KC834053 (16.4%) and Uncultured Ascomycota HM240001 (14.2%) in Oliveira Stream, and *Articulospora tetracladia* KP234360 (29.7%), *Varicosporium elodeae* JX981463 (10.9%) and *F*. *saccata* KC834053 (7.0%) in Boss Brook (S3 Table). Among active reads, other major contributors included OTUs with close hits to Basidiomycete sp. EU819529 (35.9%), *Lunulospora curvula* JX089527 (22.7%) and *Alternaria alternata* KP985749 (16.7%) in Oliveira Stream, and *Articulospora tetracladia* KP234360 (24.6%) and *Varicosporium elodeae* JX981463 (20.9%) in Boss Brook (S3 Table).

**Fig 3:**
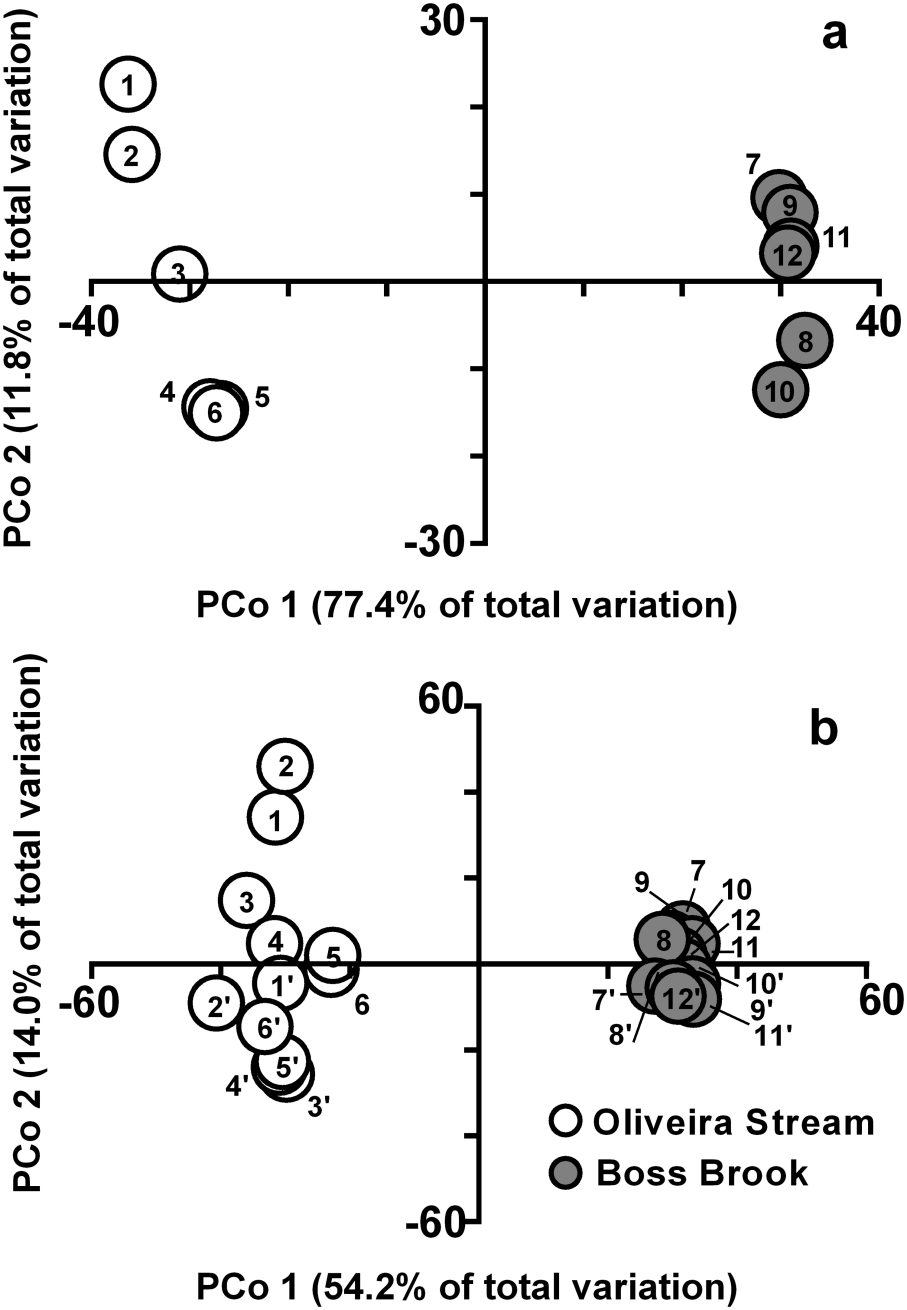
Principal coordinates (PCo) analysis for the ordination of fungal communities. Ordination was based on morphospecies according to stream (Oliveira Stream versus Boss Brook) and leaf area (0.6 to 13.6 cm^2^) (a), and based on OTUs with hits for fungi according to stream, leaf area and type of community (total versus active) (b). In a) and b) (total communities): 1 and 7, 0.6 cm^2^; 2 and 8, 1.1 cm^2^; 3 and 9, 2.3 cm^2^; 4 and 10, 4.5 cm^2^; 5 and 11, 9.0 cm^2^; 6 and 12, 13.6 cm^2^. In b), for active communities: 1’ and 7’, 0.6 cm^2^; 2’ and 8’, 1.1 cm^2^; 3’ and 9’, 2.3 cm^2^; 4’ and 10’, 4.5 cm^2^; 5’ and 11’, 9.0 cm^2^; 6’ and 12’, 13.6 cm^2^.

Principal coordinates analyses indicated that the factor stream significantly affected aquatic fungal community structure based on morphospecies or OTUs (PERMANOVA, P = 0.001 and P = 0.002, respectively) (Fig 3). Leaf area did not significantly affect community structure for morphospecies (PERMANOVA, P = 0.60) (Fig 3a), but a significant effect was found when OTUs were analysed (PERMANOVA, P = 0.001) (Fig 3b). In addition, metabarcoding revealed a significant effect for the type of community (total versus active) (PERMANOVA, P = 0.009), as well as for interactions between stream and leaf area (PERMANOVA, P = 0.001) and stream and the type of community (PERMANOVA, P = 0.02).

### Relationship between fungal diversity and leaf area

**Fig 4:**
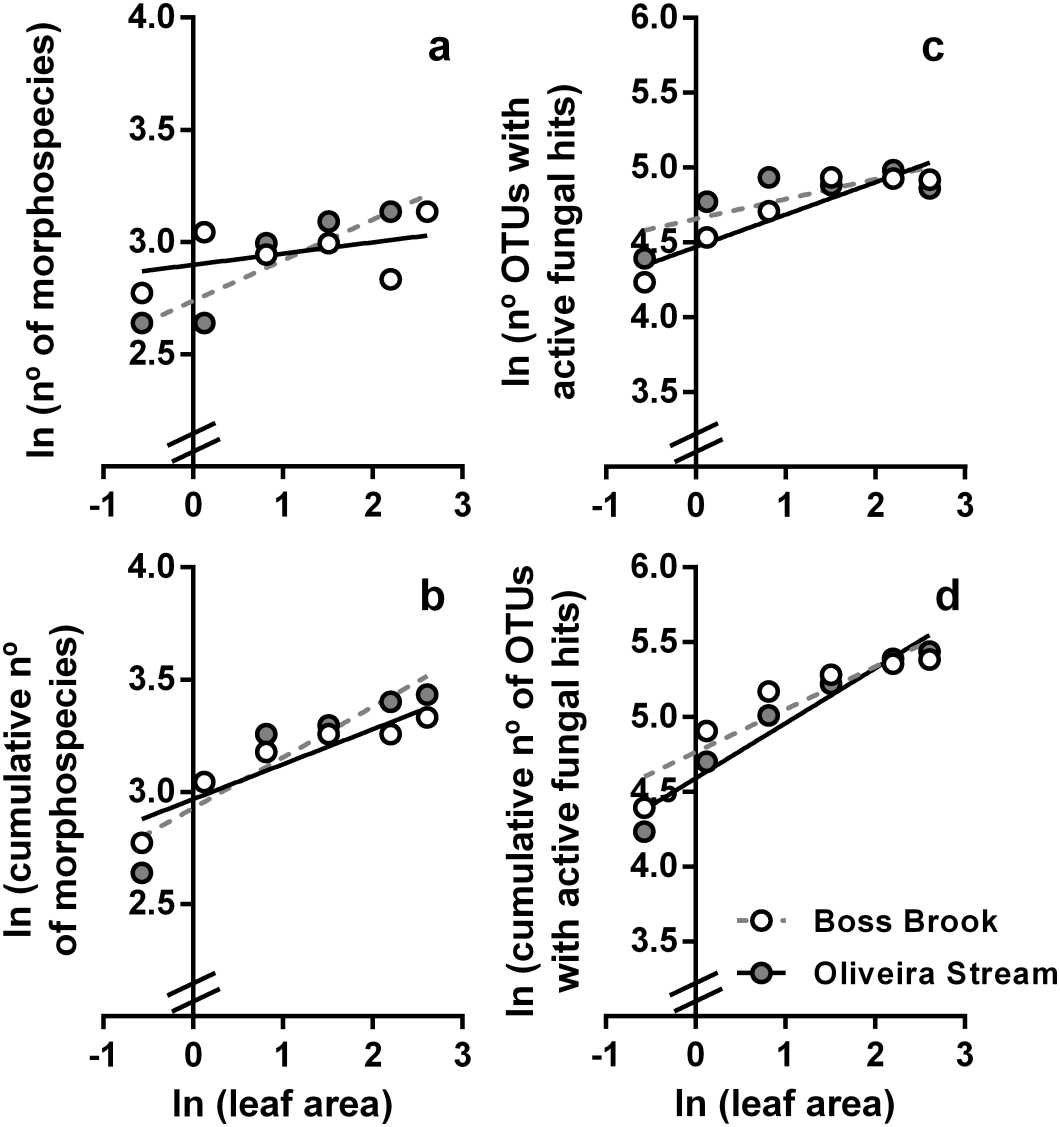
Relationship between ln (number of aquatic fungi) and ln (leaf area in cm^2^). Number of aquatic fungi was based on: morphospecies (a), cumulative number of morphospecies (b), number of OTUs with hits for active fungal taxa (c), and cumulative number of OTUs with hits for active fungal taxa (d), for alder leaves submerged in Oliveira Stream and Boss Brook, n = 6. The details of model parameters and function fits are in Table 1.

The number of species appeared to approach an upper limit within the range of leaf disk areas chosen and the visual inspection suggests a rapidly declining decrease of additional species with the sampling effort (Fig 4). We opted to use the power curve to establish the relationship between the number of fungal taxa and leaf area to assess z values, because the power function is the one most commonly used. Its use allows easier comparisons of our data with data from literature [4]. But all three models (exponential, power and logistic curves) discussed in Scheiner [4] gave good fits with our data (S4 and S5 Tables), with the exception of the relationship between Oliveira Stream morphospecies and leaf area, for which no fit was possible with the logistic function.

The rate at which the ln (number of morphospecies increased with ln (leaf area) (taxa-area curve, TAR, Fig 4a) and the rate of taxa accumulation (taxa-accumulation curve, TAC, Fig 4b) (Table 1) (z values) were ca. 4 and 2 times higher for Boss Brook than for Oliveira Stream (0.18 versus 0.05, and 0.23 versus 0.16, respectively). Ln (number of taxa) significantly increased with ln (leaf area) in Boss Brook (linear regression, r^2^ = 0.86, P = 0.007), but not in Oliveira Stream (linear regression, r^2^ = 0.21, P = 0.4) (Table 1). On the other hand, the ln (cumulative number of morphospecies) increased significantly with the ln (leaf area) in both streams (linear regression, r^2^ = 0.86, P = 0.008, for both comparisons) (Fig 4b, Table 1).

**Table 1:** Model parameters of the relationship between ln (fungal taxa) and ln (leaf area) (Fig 4).

	Stream	Parameter	Parameter value	r^2^	P
Morphospecies	Oliveira Stream	z	0.050	0.21	0.4
		c	2.90		
Morphospecies	Boss Brook	z	0.18	0.86	0.007
		c	2.74		
Cumulative morphospecies	Oliveira Stream	z	0.16	0.86	0.007
		c	2.97		
Cumulative morphospecies	Boss Brook	z	0.23	0.86	0.008
		c	2.93		
OTUs	Oliveira Stream	z	0.22	0.87	0.006
		c	4.47		
OTUs	Boss Brook	z	0.13	0.58	0.08
		c	4.66		
Cumulative OTUs	Oliveira Stream	z	0.37	0.95	0.001
		c	4.59		
Cumulative OTUs	Boss Brook	z	0.29	0.85	0.009
		c	4.76		

For details on equation, see Materials and methods, Eq 3.

The opposite trend was found for fungal diversity estimated through OTUs with hits for active fungal taxa; z values derived from TAR and from TAC curves (Fig 4c and 4d) were approximately doubled for Oliveira Stream (0.22 versus 0.13 and 0.37 versus 0.29, for TAR and TAC, respectively). The ln (number of OTUs with hits for active fungi) significantly increased with the ln (leaf area) in Oliveira Stream (linear regression, P = 0.006, r^2^ = 0.87), but not in Boss Brook (linear regression, P = 0.08, r^2^ = 0.58) (Fig 4c, Table 1). Conversely, ln (cumulative number of OTUs with hits for active fungi) increased significantly with ln (leaf area in Oliveira Stream) (linear regression, r^2^ = 0.95, P = 0.001) and Boss Brook (linear regression, r^2^ = 0.85, P = 0.009) (Fig 4d, Table 1).

## Discussion

As hypothesized, larger leaf areas in streams tended to harbour more fungal species. Our results provide evidence that fungi display spatial turnover in streams, but also that our perception of “global” species in a given stream is strongly affected by sampling effort. The most rapid increase of fungal diversity occurred at very small leaf areas, suggesting that the number of co-existing species quickly approaches a plateau (Fig 4). However, these findings were inconsistent between sampling sites and detection approaches; when diversity assessments were based on morphospecies, taxa richness increased with leaf area in Boss Brook, but not in Oliveira Stream, while the opposite was found with metabarcoding of cDNA (Fig 4). Taxa-area curves were generated using RNA-based OTUs, since RNA is much less likely to occur and be stable outside a metabolically active cell; thus any OTU based on cDNA is therefore likely to originate from an active taxon [30]. On the other hand, the cumulative number of taxa increased in both streams using either method (Fig 4). This provides further support for the proposal that taxa-area curves and taxa-accumulation curves are not equivalent [47, 48]. In both streams, leaves seemed to be in a similar stage of decomposition, and shared similar dynamics of fungal sporulation and diversity, but higher fungal biomasses were found in Boss Brook, which may account for the differences in the taxa-area curves in the two streams (Fig 1).

Z values of taxa-area curves varied between 0.05 and 0.22 for Oliveira Stream and 0.13 and 0.18 for Boss Brook, respectively, suggesting in both cases a low to intermediate rate of change or species turnover with increasing leaf area (Table 1) [7, 8]. These estimates are similar to values in two other studies on taxa-area relationships for aquatic hyphomycetes, i.e., on leaves (z = 0.11 and 0.14, for oak and maple, respectively, Germany [28]) or on wood blocks (z = 0.21, UK [26]) (S3 Fig). We hypothesised that the metabarcoding approach would reveal steeper z values, due to its greater resolution of diversity [8, 12]. This appears to be the case for Oliveira Stream, but not for Boss Brook (Fig 4). Our sampling strategy and analyses may have contributed to these inconsistent findings. Scheiner [4] defined 6 types of species-area curves based on the combination of 4 sampling schemes and data evaluation: i) strictly nested quadrats (Type I); ii) quadrats arrayed in a contiguous grid (Type II); iii) quadrats arrayed in a regular but noncontiguous grid (Type III) or iv) areas of varying size, often islands (Type IV) [4]. Type II and Type III curves are further sub-divided in a) and b), depending on whether or not sampling is based on spatially explicit methods [4]. Gray et al. [47] contended that 5 of those types depict “species-accumulation curves”, and that the term “species-area curve” should be reserved for data collected from true islands. By analysing a comprehensive dataset, Matthews et al. [48] showed that z values of species-area curves and species-accumulation curves could vary substantially within the same data set; this was also observed in the current study. Higher z values were found when the cumulative number of taxa was plotted against leaf area. In addition, in both streams, metabarcoded cumulative diversity exhibited steeper slopes than cumulative diversity based on morphospecies, as initially hypothesized (Fig 4, Table 1). This emphasizes the crucial importance of the choice of how species-area curves are constructed and the detection / identification approaches [47, 48]. Microbial taxa-area relationships with higher z values have been found in Type II curves (diversity of soil prokaryotes in quadrats arrayed in a contiguous grid [49], 0.42<z<0.47) or Type IV curves (island type or discrete areas of increasing size [7, 50], 0.25<z<0.29) or by using a much larger sampling scale (for water ecosystem types [9], 0.3<z<0.5), but the authors did not explicitly state whether z values were derived through taxa-area or taxa-accumulation curves. A complicating factor in streams is the possibility that individual leaves may be in close proximity (leaf pack accumulations), which will facilitate transfer of fungal propagules among substrates or between substrates and stream water. Both processes would tend to flatten the relationship between taxon richness and resource area in streams.

Stream identity was the major factor structuring fungal communities based on spore morphology and metabarcoding (Fig 3). In addition, leaf area significantly affected the structure of fungal communities assessed through metabarcoding. Next generation sequencing (NGS) using ITS markers can detect differences at the intraspecific/genotypic level [31], which may have accounted for the differentiation of communities based on leaf areas. To our knowledge our study is the first to use NGS to assess the relationship between taxa and resource area for aquatic fungi. Research with other fungal groups (soil fungi) employing NGS has challenged the conceptual dogma that for microbes “everything is everywhere” [5], increasing the chances of finding endemic, as well as rare or cryptic taxa and providing evidence of a significant spatial turnover within fungal communities [51–53]. There is recent evidence from NGS surveys that some fungi may have restricted and/or patchy distributions while others are clearly widespread [53]. For instance, more generalist fungi, such as saprotrophs and plant pathogens had broader distribution ranges than specialist species, such as ectomycorrhizal and arbuscular mycorrhizal root symbionts [53]. However, to take full advantage of the higher resolution and greater sensitivity of NGS, care has to be taken to use consistent spatial scales [54], sampling efforts and strategies [47, 55, 56], all of which have been shown to strongly influence z values [9]. NGS is also prone to other methodological biases, such as the sequencing depth (Fig 2), which can strongly affect the taxa-area relationship [9, 13, 57, 58].

Contrary to our expectations, we did not find greater OTU numbers in total (DNA) vs. active (RNA) communities (S3 Table). In addition, the composition of the two communities differed significantly and RNA based OTUs were not a subset of DNA based OTUs (Fig 3, S3 Table). Baldrian et al. [30] found similar discrepancies in a study on forest soil fungi, and concluded that several highly active taxa show low abundance or were absent in the DNA pool. Thus, the presence of exclusive OTUs in the RNA pool, as also found in our study, suggest that DNA-based surveys may miss considerable portions of active microbial populations and RNA-based surveys may be more meaningful. Even so, dominant OTUs did not differ significantly; OTUs with hits for Ascomycota members dominated both pools, and OTUs with hits for *Articulospora tetracladia* EU998923 dominated total and active reads in both streams in our study (S3 Table). Baldrian et al. [30] also assigned exocellulase activities to taxa and found further discrepancies between RNA and enzyme pools. When the primary goal is to establish the geographical distribution of different taxa, DNA based NGS seems appropriate. To identify major contributors to ecosystem processes, a combination of transcriptomics and proteomics is likely to yield more appropriate results. It must be kept in mind, however, that metabolic activities of different fungal taxa wax and wane, influenced by successional stage and season.

A more comprehensive approach in future studies should include fungal taxa in different types of substrata (i.e. different leaf species, mixtures of leaves, wood, twigs) and at larger spatial (i.e. streams from source to mouth, or several streams per geographic location) and temporal (different stages of decomposition, seasons) scales, and include DNA, RNA and protein analyses [30, 59]. Pronounced environmental heterogeneity should favour higher fungal diversity, especially if species or strains are not randomly distributed and display substrate preferences [25] and are affected by environmental and anthropogenic factors (e.g. temperature [23], pH [60], conductivity [61], altitude [23], nutrient loads [62]). Taxa-area relationships slopes are generally steeper in heterogeneous habitats [9, 63, 64]. In marine bacteria, slopes were steeper in highly heterogeneous sediments and coastal environments than in the more monotonous, pelagic zone [9].

Overall, our results suggest a positive relationship between decomposer fungal diversity and surveyed leaf area in streams. Steeper slopes in the taxa-area curves in both streams based on NGS were obtained when using taxa-accumulation curves. Our findings do not allow us to draw final conclusions concerning local to global richness ratios, but in a recent literature survey a total of approximately 335 aquatic hyphomycete morphospecies were reported to occur in world freshwaters [21]. In the current study we were able to find as much as 10% of this currently reported global diversity, and ca. 16 and 18% of the diversity reported from the east coast of Canada and Portugal, respectively [21]. However, we must take into account that there are undiscovered species in almost every taxonomic survey or species inventory. Fenchel et al. [65] also reported about 10% of the estimated global diversity of free-living ciliates in local samples and therefore concluded that “everything is (almost) everywhere”. However, a closer look revealed this conclusion to be questionable since true protist diversity remains largely unknown [65]. This may also be true for aquatic fungi. Recent global sequencing efforts in soils have revealed a large amount of hidden fungal diversity and a scarcity of globally distributed OTUs (e.g. [52]). Aquatic habitats, in particular streams, are still largely unexplored. In fact, in our study 32.6% of OTUs could not be assigned to any known fungal species, suggesting the presence of unknown fungal lineages. Increasing use of NGS in biodiversity surveys promises greater accuracy in revealing diversity patterns of stream-dwelling decomposer fungi, taxa-area relationships, local to global diversity ratios and will put aquatic fungi on the map. To that end, both morphological and molecular data will be necessary, which will also shed light on the unexplored diversity of aquatic fungi in general.

## Supporting information

S1 Fig ITS PCR and RT-PCR products for each treatment. Products were generated from DNA and RNA pools from leaf disks that were immersed at Boss Brook. M) DNA ladder BenchTop 100 bp (Promega), 1) DNA 0.6 cm^2^, 2) DNA 1.1 cm^2^, 3) DNA 2.3 cm^2^, 4) DNA 4.5 cm^2^, 5) DNA 9.0 cm^2^, 6) DNA 13.6 cm^2^, 7) DNA negative control, 8) RNA 0.6 cm^2^, 9) RNA 1.1 cm^2^, 10) RNA 2.3 cm^2^, 11) RNA 4.5 cm^2^, 12) RNA 9.0 cm^2^, 13) RNA 13.6 cm^2^, 14) RNA negative control, 15) cDNA 0.6 cm^2^, 16) cDNA 1.1 cm^2^, 17) cDNA 2.3 cm^2^, 18) cDNA 4.5 cm^2^, 19) cDNA 9.0 cm^2^, 20) cDNA 13.6 cm^2^ and 21) cDNA negative control.
(TIF)

S2 Fig N° of reads on alder leaf disks. N° of reads with hits for total (DNA) and active OTUs (RNA) on leaf disks submerged in Oliveira Stream and Boss Brook, n = 6.
(TIF)

S3 Fig Z values (+ SE) obtained in studies reporting taxa-area relationships for aquatic hyphomycetes (morphospecies). Narrator Brook [26], Ibach [27], Oliveira Stream (current study) and Boss Brook (current study). The dashed line represents the z value calculated by using the data from all studies and the power function (Eq 3).
(TIF)

S1 Table Maximum % contribution of each aquatic fungal taxon. Maximum % contribution was based on morphospecies sporulating on leaf disks submerged in Oliveira Stream and Boss Brook.
(DOCX)

S2 Table Model parameters estimated for rarefaction curves. Rarefaction curves were based on alpha diversity measures (observed OTUs, Chao-1 richness index and Shannon diversity index) versus the sampling effort (sequencing depth). Michaelis-Menten models gave the best fits for these relationships (Eq 2).
(DOCX)

S3 Table List of OTUs with hits for fungi (total for DNA and active for RNA data). The list includes closest NCBI database match and respective % identity and accession number and maximum % contribution of each OTU, based on the number of reads, on leaf disks submerged in Oliveira Stream and Boss Brook. *OTUs with close hits with aquatic hyphomycete sequences.
(DOCX)

S4 Table Model parameters of the relationship between fungal taxa diversity and leaf area (Fig 4a and 4c) in TAR curves. The exponential (EC) (Eq 4) and the logistic (LC) models (Eq 5) [4] were used.
(DOCX)

S5 Table Model parameters of the relationship between fungal taxa diversity and leaf area (Fig 4b and 4d) in TAC curves. The exponential (EC) (Eq 4) and the logistic (LC) models (Eq 5) [4] were used.
(DOCX)

## References

[1] Arrhenius O. Species and area. J Ecol. 1921; 9:95–99.

[2] Rosenzweig ML. Species diversity in space and time. Cambridge University Press; 1995.

[3] MacArthur RH. Patterns of species diversity. Biol Rev. 1965; 40:510–533.

[4] Scheiner SM Six types of species-area curves. Glob Ecol Biogeogr. 2003; 12:441–447.

[5] Baas Becking LGM. Geobiologie of inleiding tot de milieukunde. WP Van Stockum and Zoon, The Hague; 1934.

[6] Fenchel T, Finlay BJ. The ubiquity of small species: patterns of local and global diversity. Bioscience 2004; 54:777–784.

[7] Bell T, Ager D, Song J-I, Newman JA, Thompson IP, Lilley AK, et al Larger islands house more bacterial taxa. Science. 2005; 308:1884 doi: 10.1126/science.1111318 15976296 10.1126/science.1111318

[8] Green J, Bohannan BJM. Spatial scaling of microbial biodiversity. Trends Ecol Evol. 2006; 21:501–507. doi: 10.1016/j.tree.2006.06.012 16815589 10.1016/j.tree.2006.06.012

[9] Zinger L, Boetius A, Ramette A. Bacterial taxa—area and distance—decay relationships in marine environments. Mol Ecol. 2014; 23:954–964. doi: 10.1111/mec.12640 24460915 10.1111/mec.12640 PMC4230465

[10] Taylor JW, Turner E, Townsend JP, Dettman JR, Jacobson D. Eukaryotic microbes, species recognition and the geographic limits of species: examples from the kingdom Fungi. Phil Trans R Soc B. 2006; 361: 1947–1963. doi: 10.1098/rstb.2006.1923 17062413 10.1098/rstb.2006.1923 PMC1764934

[11] Bärlocher F. Freshwater fungal communities In: Deighton J, Oudemans P, White J, editors. The fungal community: its organization and role in the ecosystem. 3rd ed Taylor and Francis, CRC Press; 2005 pp. 39–59.

[12] Bärlocher F. Molecular approaches promise a deeper and broader understanding of the evolutionary ecology of aquatic hyphomycetes. J N Am Benthol Soc. 2010; 29:1027–1041.

[13] Woodcock S, Curtis TP, Head IM, Lunn M, Sloan WT. Taxa-area relationships for microbes: the unsampled and the unseen. Ecol Lett. 2006; 9:805–812. doi: 10.1111/j.1461-0248.2006.00929.x 16796570 10.1111/j.1461-0248.2006.00929.x

[14] Azovsky AI. Size-dependent species-area relationships in benthos: is the world more diverse for microbes? Ecography 2002; 25:273–282.

[15] Finlay BJ. Global dispersal of free-living microbial eukaryote species. Science. 2002; 296:1061–1063. doi: 10.1126/science.1070710 12004115 10.1126/science.1070710

[16] Green JL, Holmes AJ, Westoby M, Oliver I, Briscoe D, Dangerfield M, et al Spatial scaling of microbial eukaryote diversity. Nature. 2004; 432:747–750. doi: 10.1038/nature03034 15592411 10.1038/nature03034

[17] Horner-Devine MC, Lage M, Hughes JB, Bohannan BJM. A taxa—area relationship for bacteria. Nature. 2004; 432:750–753. doi: 10.1038/nature03073 15592412 10.1038/nature03073

[18] Suberkropp K. Microorganisms and organic matter decomposition. Springer; 1998.

[19] Nikolcheva LG, Cockshutt AM, Bärlocher F. Determining diversity of freshwater fungi on decaying leaves: comparison of traditional and molecular approaches. Appl Environ Microbiol. 2003; 69:2548–2554. doi: 10.1128/AEM.69.5.2548-2554.2003 12732520 10.1128/AEM.69.5.2548-2554.2003 PMC154547

[20] Gulis V, Marvanová L, Descals E. An illustrated key to the common temperate species of aquatic hyphomycetes In: Graça MAS, Bärlocher F, Gessner MO, editors. Methods to study litter decomposition a practical guide. Springer; 2005 pp. 153–168.

[21] Duarte S, Bärlocher F, Pascoal C, Cássio F. Biogeography of aquatic hyphomycetes: current knowledge and future perspectives. Fungal Ecol. 2016; 19:169–181.

[22] Bärlocher F. Reproduction and dispersal in aquatic hyphomycetes. Mycoscience. 2009; 50:3–8.

[23] Bärlocher F, Stewart M, Ryder DS. Analyzing aquatic fungal communities in Australia: impacts of sample incubation and geographic distance of streams. Czech Mycol. 2011; 63:113–132.

[24] Duarte S, Cássio F, Pascoal C. Environmental drivers are more important for structuring fungal decomposer communities than the geographic distance between streams. Limnetica. 2017; accepted.

[25] Charcosset J-Y, Gardes M. Infraspecific genetic diversity and substrate preference in the aquatic hyphomycete *Tetrachaetum elegans*. Mycol Res. 1999; 100:736–742.

[26] Sanders PF, Anderson JM. Colonization of wood blocks by aquatic hyphomycetes. Trans Br Mycol Soc. 1979; 73:103–107.

[27] Bärlocher F. Leaf-eating invertebrates as competitors of aquatic hyphomycetes. Oecologia. 1980; 47:303–306. doi: 10.1007/BF00398521 28309079 10.1007/BF00398521

[28] Bärlocher F, Schweizer M. Effects of leaf size and decay rate on colonization by aquatic hyphomycetes. Oikos. 1983; 41:205–210.

[29] Bärlocher F. Aquatic hyphomycetes in a changing environment. Fungal Ecol. 2016; 19:14–27.

[30] Baldrian P, Kolařík M, Štursová M, Kopecký J, Válašková V, Větrovský T, et al Active and total microbial communities in forest soil are largely different and highly stratified during decomposition. ISME J. 2012; 6:248–258. doi: 10.1038/ismej.2011.95 21776033 10.1038/ismej.2011.95 PMC3260513

[31] Duarte S, Bärlocher F, Trabulo J, Cássio F, Pascoal C. Stream-dwelling fungal decomposer communities along a gradient of eutrophication unraveled by 454 pyrosequencing. Fungal Divers. 2015; 70:127–148.

[32] Dunck B, Lima-Fernandes E, Cássio F, Cunha A, Rodrigues L, Pascoal C. Responses of primary production, leaf litter decomposition and associated communities to stream eutrophication. Environ Pollut. 2015; 202:32–40. doi: 10.1016/j.envpol.2015.03.014 25797823 10.1016/j.envpol.2015.03.014

[33] Lima-Fernandes E, Fernandes I, Pereira A, Geraldes P, Cássio F, Pascoal C. Eutrophication modulates plant-litter diversity effects on litter decomposition in streams. Freshw Sci. 2015; 34:31–41.

[34] Nikolcheva LG, Bärlocher F. Seasonal and substrate preferences of fungi colonizing leaves in streams: traditional versus molecular evidence. Environ Microbiol. 2005; 7:270–280. doi: 10.1111/j.1462-2920.2004.00709.x 15658994 10.1111/j.1462-2920.2004.00709.x

[35] Grimmett IJ, Smith KA, Bärlocher F. Tar-spot infection delays fungal colonization and decomposition of maple leaves. Freshw Sci. 2012; 31:1088–1095.

[36] Wurzbacher C, Grimmett IJ, Bärlocher F Metabarcoding-based fungal diversity on coarse and fine particulate organic matter in a first-order stream in Nova Scotia, Canada. F1000Res. 2015; 4:1378 doi: 10.12688/f1000research.7359.2 26918122 10.12688/f1000research.7359.1 PMC4755416

[37] Gessner MO. Ergosterol as measure of fungal biomass In: Graça MAS, Bärlocher F, Gessner MO, editors. Methods to study litter decomposition a practical guide. Springer; 2005 pp. 189–195.

[38] White TJ, Bruns T, Lee S, Taylor JW Amplification and direct sequencing of fungal ribosomal RNA genes for phylogenetics In: Innis MA, Gelfand DH, Sninsky JJ, White TJ, editors. PCR protocols: a guide to methods and applications. Academic Press, Inc.; 1990 pp. 315–322.

[39] Gardes M, Bruns T. ITS primers with enhanced specificity for basidiomycetes—application to the identification of mycorrhizae and rusts. Mol Ecol. 1993; 2:113–118. 8180733 10.1111/j.1365-294x.1993.tb00005.x

[40] Fernandes I, Pereira A, Trabulo J, Pascoal C, Cássio F, Duarte S. Microscopy- or DNA-based analyses: Which methodology gives a truer picture of stream-dwelling decomposer fungal diversity? Fungal Ecol. 2015; 18:130–134.

[41] Edgar RC, Haas BJ, Clemente JC, Quince C, Knight R. UCHIME improves sensitivity and speed of chimera detection. Bioinform. 2011; 27:2194–2200. 10.1093/bioinformatics/btr381 PMC3150044 21700674

[42] Caporaso JG, Kuczynski J, Stombaugh J, Bittinger K, Bushman FD, Costello EK, et al QIIME allows analysis of high-throughput community sequencing data. Nat Methods 2010; 7:335–336. doi: 10.1038/nmeth.f.303 20383131 10.1038/nmeth.f.303 PMC3156573

[43] DeSantis TZ, Hugenholtz P, Larsen N, Rojas M, Brodie EL, Keller K, et al Greengenes, a chimera-checked 16S rRNA gene database and workbench compatible with ARB. Appl Environ Microbiol. 2006; 72:5069–5072. doi: 10.1128/AEM.03006-05 16820507 10.1128/AEM.03006-05 PMC1489311

[44] Zar JH. Biostatistical analysis. 5th ed Pearson Prentice-Hall; 2010.

[45] Motulsky HJ, Christopoulos A. Fitting models to biological data using linear and nonlinear regression. A practical guide to curve fitting, GraphPad Software Inc., San Diego, CA, U.S.A., www.graphpad.com; 2003.

[46] Anderson MJ. A new method for non-parametric multivariate analysis of variance. Austral Ecol. 2001; 26:32–46.

[47] Gray JS, Ugland KI, Lambshead J. Species accumulation and species area curves—a comment on Scheiner (2003). Glob Ecol Biogeogr. 2004; 13:473–476.

[48] Matthews TJ, Triantis KA, Rigal F, Borregaard MK, Guilhaumon F, Whittakey RJ. Island species—area relationships and species accumulation curves are not equivalent: an analysis of habitat island datasets. Glob Ecol Biogeogr. 2016; 25:607–618.

[49] Noguez AM, Arita HT, Escalante AE, Forney LJ, García-Oliva F, Souza V. Microbial macroecology: highly structured prokaryotic soil assemblages in a tropical deciduous forest. Glob Ecol Biogeogr. 2005; 14:241–248.

[50] Van der Gast CJ, Lilley AK, Ager D, Thompson IP. Island size and bacterial diversity in an archipelago of engineering machines. Environ Microbiol. 2005; 7:1220–1226. doi: 10.1111/j.1462-2920.2005.00802.x 16011759 10.1111/j.1462-2920.2005.00802.x

[51] Peay KG, Bruns TD, Kennedy PG, Bergemann SE, Garbelotto M. A strong species—area relationship for eukaryotic soil microbes: island size matters for ectomycorrhizal fungi. Ecol Lett. 2007; 10:470–480. doi: 10.1111/j.1461-0248.2007.01035.x 17498146 10.1111/j.1461-0248.2007.01035.x

[52] Meiser A, Bálint M, Schmitt I. Meta-analysis of deep-sequenced fungal communities indicates limited taxon sharing between studies and the presence of biogeographic patterns. New Phytol. 2014; 201:623–635. doi: 10.1111/nph.12532 24111803 10.1111/nph.12532

[53] Tedersoo L, Bahram M, Põlme S, Kõljalg U, Yorou NS, Wijesundera R, et al Global diversity and geography of soil fungi. Science. 2014; 346:6213. 10.1126/science.1256688 25430773

[54] Steinbauer MJ, Dolos K, Reineking B, Beierkuhnlein C. Current measures for distance decay in similarity of species composition are influenced by study extent and grain size. Glob Ecol Biogeogr. 2012; 21:1203–1212.

[55] Cardoso P, Borges PAV, Veech JA. Testing the performance of beta diversity measures based on incidence data: the robustness to undersampling. Divers Distrib. 2009; 15:1081–1090.

[56] Dengler J. Which function describes the species-area relationship best? A review and empirical evaluation. J Biogeogr. 2009; 36:728–744.

[57] Tedersoo L, Nilsson RH, Abarenkov K, Jairus T, Sadam A, Saar I, et al 454 pyrosequencing and Sanger sequencing of tropical mycorrhizal fungi provide similar results but reveal substantial methodological biases. New Phytol. 2010; 188:291–301. doi: 10.1111/j.1469-8137.2010.03373.x 20636324 10.1111/j.1469-8137.2010.03373.x

[58] Lindhal BJ, Nilsson RH, Tedersoo L, Abarenkov K, Carlsen T, Kjøller R, et al Fungal community analysis by high-throughput sequencing of amplified markers—a user’s guide. New Phytol. 2013; 199:288–299. doi: 10.1111/nph.12243 23534863 10.1111/nph.12243 PMC3712477

[59] Grimmett IJ, Shipp KN, MacNeil A, Bärlocher F. Does the growth rate hypothesis apply to aquatic hyphomycetes? Fungal Ecol. 2013; 6:493–500.

[60] Bärlocher F, Rosset J. Aquatic hyphomycete spora of two black forest and two swiss jura streams. Trans Br Mycol Soc. 1981; 76:479–483.

[61] Wood-Eggenschwiler S, Bärlocher F. Aquatic hyphomycetes in sixteen streams in France, Germany and Switzerland. Trans Brit Mycol Soc. 1983; 81:371–379.

[62] Duarte S, Pascoal C, Cássio F, Garabétien F, Charcosset J-Y. Microbial decomposer communities are mainly structured by trophic status in circumneutral and alkaline Streams. Appl Environ Microbiol. 2009; 75:6211–6221. doi: 10.1128/AEM.00971-09 19648371 10.1128/AEM.00971-09 PMC2753096

[63] Drakare S, Lennon JJ, Hillebrand H. The imprint of the geographical, evolutionary and ecological context on species area relationships. Ecol Lett. 2006; 9:215–227. doi: 10.1111/j.1461-0248.2005.00848.x 16958886 10.1111/j.1461-0248.2005.00848.x

[64] Soininen J, McDonald R, Hillebrand H. The distance decay of similarity in ecological communities. Ecography. 2007; 30:3–12.

[65] Fenchel T, Esteban GF, Finlay BJ. Local versus global diversity of microorganisms: cryptic diversity of ciliated protozoa. Oikos. 1997; 80:220–225.

