# Supplementary figures and images for: Taxa-area relationship of aquatic fungi on deciduous leaves
Source: PLoS One. 2017 Jul 18;12(7):e0181545. doi: 10.1371/journal.pone.0181545 (PMC5515451; doi:10.1371/journal.pone.0181545)

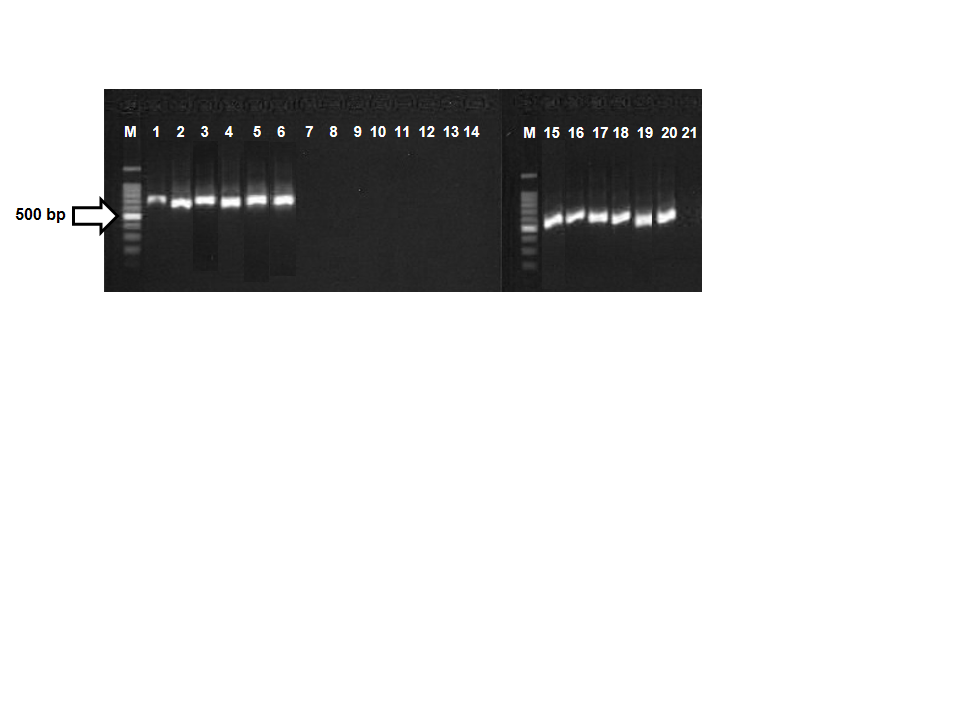

Supplement: S1 Fig — Products were generated from DNA and RNA pools from leaf disks that were immersed at Boss Brook. M) DNA ladder BenchTop 100 bp (Promega), 1) DNA 0.6 cm2, 2) DNA 1.1 cm2, 3) DNA 2.3 cm2, 4) DNA 4.5 cm2, 5) DNA 9.0 cm2, 6) DNA 13.6 cm2, 7) DNA negative control, 8) RNA 0.6 cm2, 9) RNA 1.1 cm2, 10) RNA 2.3 cm2, 11) RNA 4.5 cm2, 12) RNA 9.0 cm2, 13) RNA 13.6 cm2, 14) RNA negative control, 15) cDNA 0.6 cm2, 16) cDNA 1.1 cm2, 17) cDNA 2.3 cm2, 18) cDNA 4.5 cm2, 19) cDNA 9.0 cm2, 20) cDNA 13.6 cm2 and 21) cDNA negative control. (TIF) [file pone.0181545.s001.tif]
