# Supplementary material for: Taxa-area relationship of aquatic fungi on deciduous leaves
Source: PLoS One. 2017 Jul 18;12(7):e0181545. doi: 10.1371/journal.pone.0181545 (PMC5515451; doi:10.1371/journal.pone.0181545)
Supplement: S1 Table — Maximum % contribution was based on morphospecies sporulating on leaf disks submerged in Oliveira Stream and Boss Brook. (DOCX) [file pone.0181545.s004.docx]

**S1 Table.** **Maximum % contribution of each aquatic fungal taxon.** Maximum % contribution was based on morphospecies sporulating on leaf disks submerged in Oliveira Stream and Boss Brook.

| Species | Oliveira Stream | Boss Brook |
| --- | --- | --- |
| *Alatospora acuminata* Ingold | 2.9 | 1.5 |
| *Alatospora pulchella* Marvanová | 3.4 | 0.3 |
| *Anguillospora filiformis* Greath. | 0.1 | 37.4 |
| *Articulospora tetracladia* Ingold | 4.0 | 36.1 |
| *Clavariopsis aquatica* De Wild. | 0.1 | 1.0 |
| *Clavatospora longibrachiata* (Ingold) Marvanová & Sv. Nilsson | 13.5 | 1.2 |
| *Culicidospora aquatica* R.H. Petersen |  | 0.5 |
| *Cylindorcarpon* sp. | 0.1 |  |
| *Dendrospora fusca* Descals & J. Webster |  | 0.1 |
| *Dendrospora* sp. |  | <0.1 |
| *Dimorphospora foliicola* Tubaki | 33.3 | 0.7 |
| *Flagellospora curvula* Ingold | 39.9 | 46.2 |
| *Aquanectria penicillioides* (Ingold) Lombard & Crous | 33.6 | 0.4 |
| *Flagellospora* sp. | 0.8 |  |
| *Fontanospora fusiramosa* Marvanová, Peter J. Fisher & Descals |  | 0.6 |
| *Fusarium* sp. |  | 0.3 |
| *Geniculospora grandis* Greath. ex Nolan |  | 0.2 |
| *Geniculospora inflata* (Ingold) Marvanová & Sv. Nilsson |  | 0.6 |
| *Goniopila monticola* (Dyko) Marvanová & Descals | 0.3 |  |
| *Heliscella stellata* (Ingold & V.J. Cox) Marvanová & Sv. Nilsson | 1.4 | 0.1 |
| *Neonectria lugdunensis* (Sacc. & Thérry) Lombard & Crous | 3.8 | 0.7 |
| *Infundibura* sp. | 2.9 |  |
| *Lemonniera aquatica* De Wild. | 0.8 | 0.3 |
| *Lemonniera terrestris* Tubaki |  | <0.1 |
| *Lunulospora curvula* Ingold | 4.1 |  |
| *Mycocentrospora clavata* S.H. Iqbal |  | 0.1 |
| *Mycocentrospora* sp. |  | 0.4 |
| *Mycofalcella calcarata* Marvanová, Om-Kalth. & J. Webster |  | 0.5 |
| *Stenocladiella neglecta* (Marvanová & Descals) Marvanová & Descals | 0.1 |  |
| *Taeniospora gracilis* Marvanová | <0.1 |  |
| *Tetrachaetum elegans* Ingold | 6.0 |  |
| *Tetracladium marchalianum* De Wild. | 0.1 |  |
| *Tetracladium setigerum* (Grove) Ingold | 0.1 |  |
| *Tricladiopsis flagelliformis* Descals |  | 0.1 |
| *Tricladium chaetocladium* Ingold | 16.5 |  |
| *Tricladium minutum* (S.H. Iqbal) Marvanová & Descals |  | 0.1 |
| *Tricladium splendens* Ingold | <0.1 |  |
| *Triscelophorus acuminatus* Nawawi | 0.6 |  |
| *Triscelophorus monosporus* Ingold | <0.1 |  |
| *Trispospermum* sp. | 0.1 |  |
| *Varicosporum elodeae* W. Kegel |  | 1.2 |
| Sigmoid 1 (50-70/2-3 µm) | 0.2 | 0.2 |
| Sigmoid 2 (10-20/1 µm) | 0.1 |  |
| Sigmoid 3 (40-50/1-1.5µm) |  | 0.1 |
| Sigmoid 4 (80-100/1.5-2µm) |  | 0.1 |
| unknown tetraradiate 1 |  | <0.1 |
| unknown tetraradiate 2 |  | 0.1 |
| Nº of morphospecies | 29 | 31 |
