# Supplementary material for: Taxa-area relationship of aquatic fungi on deciduous leaves
Source: PLoS One. 2017 Jul 18;12(7):e0181545. doi: 10.1371/journal.pone.0181545 (PMC5515451; doi:10.1371/journal.pone.0181545)
Supplement: S2 Table — Rarefaction curves were based on alpha diversity measures (observed OTUs, Chao-1 richness index and Shannon diversity index) versus the sampling effort (sequencing depth). Michaelis-Menten models gave the best fits for these relationships (Eq 2). (DOCX) [file pone.0181545.s005.docx]

**S2 Table. Model parameters estimated for rarefaction curves.** Rarefaction curves were based on alpha diversity measures (observed OTUs, Chao-1 richness index and Shannon diversity index) versus the sampling effort (sequencing depth). Michaelis-Menten models gave the best fits for these relationships (equation 2).

|  | Stream | Leaf area  (cm^2^) | Parameter | Parameter value | r^2^ |
| --- | --- | --- | --- | --- | --- |
| Observed OTUs | Oliveira Stream | 0.6 | Vmax | 193.0 | 0.98 |
|  |  | 0.6 | Km | 4602 |  |
|  |  | 1.1 | Vmax | 221.3 | 0.97 |
|  |  | 1.1 | Km | 2647 |  |
|  |  | 2.3 | Vmax | 130.6 | 0.97 |
|  |  | 2.3 | Km | 2487 |  |
|  |  | 4.5 | Vmax | 168.5 | 0.97 |
|  |  | 4.5 | Km | 2450 |  |
|  |  | 9.0 | Vmax | 218.1 | 0.98 |
|  |  | 9.0 | Km | 3358 |  |
|  |  | 13.6 | Vmax | 228.1 | 0.98 |
|  |  | 13.6 | Km | 3381 |  |
|  | Boss Brook | 0.6 | Vmax | 68.31 | 0.96 |
|  |  | 0.6 | Km | 2920 |  |
|  |  | 1.1 | Vmax | 85.89 | 0.96 |
|  |  | 1.1 | Km | 2396 |  |
|  |  | 2.3 | Vmax | 92.89 | 0.97 |
|  |  | 2.3 | Km | 2924 |  |
|  |  | 4.5 | Vmax | 94.51 | 0.96 |
|  |  | 4.5 | Km | 2362 |  |
|  |  | 9.0 | Vmax | 79.08 | 0.95 |
|  |  | 9.0 | Km | 2263 |  |
|  |  | 13.6 | Vmax | 79.51 | 0.95 |
|  |  | 13.6 | Km | 2446 |  |
| Chao-1 | Oliveira Stream | 0.6 | Vmax | 307.4 | 0.94 |
|  |  | 0.6 | Km | 2209 |  |
|  |  | 1.1 | Vmax | 302.2 | 0.96 |
|  |  | 1.1 | Km | 1376 |  |
|  |  | 2.3 | Vmax | 252.2 | 0.93 |
|  |  | 2.3 | Km | 2823 |  |
|  |  | 4.5 | Vmax | 291.3 | 0.94 |
|  |  | 4.5 | Km | 2317 |  |
|  |  | 9.0 | Vmax | 365.8 | 0.95 |
|  |  | 9.0 | Km | 2527 |  |
|  |  | 13.6 | Vmax | 343.4 | 0.95 |
|  |  | 13.6 | Km | 1965 |  |
|  | Boss Brook | 0.6 | Vmax | 203.5 | 0.87 |
|  |  | 0.6 | Km | 5570 |  |
|  |  | 1.1 | Vmax | 172.3 | 0.91 |
|  |  | 1.1 | Km | 2991 |  |
|  |  | 2.3 | Vmax | 155.0 | 0.90 |
|  |  | 2.3 | Km | 2285 |  |
|  |  | 4.5 | Vmax | 190.5 | 0.90 |
|  |  | 4.5 | Km | 3104 |  |
|  |  | 9.0 | Vmax | 195.5 | 0.89 |
|  |  | 9.0 | Km | 3961 |  |
|  |  | 13.6 | Vmax | 173.4 | 0.89 |
|  |  | 13.6 | Km | 3412 |  |
| Shannon | Oliveira Stream | 0.6 | Vmax | 2.625 | 0.94 |
|  |  | 0.6 | Km | 8.617 |  |
|  |  | 1.1 | Vmax | 4.228 | 0.96 |
|  |  | 1.1 | Km | 9.520 |  |
|  |  | 2.3 | Vmax | 2.783 | 0.95 |
|  |  | 2.3 | Km | 9.157 |  |
|  |  | 4.5 | Vmax | 3.314 | 0.95 |
|  |  | 4.5 | Km | 9.512 |  |
|  |  | 9.0 | Vmax | 2.778 | 0.91 |
|  |  | 9.0 | Km | 10.60 |  |
|  |  | 13.6 | Vmax | 3.057 | 0.93 |
|  |  | 13.6 | Km | 11.39 |  |
|  | Boss Brook | 0.6 | Vmax | 1.304 | 0.89 |
|  |  | 0.6 | Km | 8.836 |  |
|  |  | 1.1 | Vmax | 2.011 | 0.92 |
|  |  | 1.1 | Km | 7.283 |  |
|  |  | 2.3 | Vmax | 2.249 | 0.94 |
|  |  | 2.3 | Km | 6.509 |  |
|  |  | 4.5 | Vmax | 2.084 | 0.93 |
|  |  | 4.5 | Km | 8.446 |  |
|  |  | 9.0 | Vmax | 1.973 | 0.90 |
|  |  | 9.0 | Km | 7.437 |  |
|  |  | 13.6 | Vmax | 2.019 | 0.91 |
|  |  | 13.6 | Km | 6.395 |  |
