# Supplementary material for: Taxa-area relationship of aquatic fungi on deciduous leaves
Source: PLoS One. 2017 Jul 18;12(7):e0181545. doi: 10.1371/journal.pone.0181545 (PMC5515451; doi:10.1371/journal.pone.0181545)
Supplement: S3 Table — The list includes closest NCBI database match and respective % identity and accession number and maximum % contribution of each OTU, based on the number of reads, on leaf disks submerged in Oliveira Stream and Boss Brook. *OTUs with close hits with aquatic hyphomycete sequences. (DOCX) [file pone.0181545.s006.docx]

**S3 Table.** **List of OTUs with hits for fungi (total for DNA and active for RNA data).** The list includes closest NCBI database match and respective % identity and accession number and maximum % contribution of each OTU, based on the number of reads, on leaf disks submerged in Oliveira Stream and Boss Brook. *OTUs with close hits with aquatic hyphomycete sequences.

|  |  |  | Oliveira Stream |  | Boss Brook |  |
| --- | --- | --- | --- | --- | --- | --- |
| Closest match GenBank (NCBI) | %ID | Accession nº | Total | Active | Total | Active |
| *Articulospora tetracladia* strain CCM F-14298* | 99 | EU998923 | 84.3 | 57.0 | 72.5 | 78.5 |
| *Articulospora tetracladia* strain CCM F-14299* | 95 | KP234360 | 10.1 | 0.7 | 29.7 | 24.6 |
| *Varicosporium elodeae* strain WA0000019152* | 98 | JX981463 | <0.1 | 1.2 | 10.9 | 20.9 |
| *Articulospora tetracladia* strain CCM F-14298* | 99 | EU998923 | 0.2 | <0.1 | 5.1 | 5.7 |
| *Flagellospora saccata* strain CCM F-39994* | 96 | KC834053 | 16.4 | 1.2 | 7.0 | 2.1 |
| *Lunulospora curvula* isolate UMB-499.09* | 99 | JX089527 | 3.9 | 22.7 |  | <0.1 |
| Basidiomycete sp. RT00050 | 99 | EU819529 | 5.0 | 35.9 |  | <0.1 |
| *Articulospora tetracladia* strain CCM F-10113* | 99 | KP234360 | 0.1 | 4.1 | 1.6 | 4.8 |
| *Alternaria alternata* strain BS14 | 100 | KP985749 | 8.9 | 16.7 | 0.9 | 0.8 |
| Uncultured fungus clone Singleton_(137-1123_0552) | 99 | FJ778362 | 0.2 | <0.1 | 0.6 | 2.7 |
| *Articulospora tetracladia* strain CCM F-10113* | 94 | KP234360 | 9.9 | 12.1 | 0.5 | 0.4 |
| Helotiales sp. 859 | 99 | GU934595 |  | 0.1 | 4.1 | 5.0 |
| Uncultured Ascomycota clone 476 | 95 | HM240001 | 14.2 | 11.8 | 0.1 | <0.1 |
| Helotiales sp. 1 PRJ-2011 | 90 | JN225950 | 45.7 | 9.3 | <0.1 | <0.1 |
| *Articulospora tetracladia* strain CCM F-10113* | 97 | KP234360 | 0.4 | 0.5 | 0.7 | 0.6 |
| Uncultured fungus clone 109A76754 | 100 | JX388477 | 3.9 | 1.8 | 1.1 | 1.4 |
| *Clavariopsis aquatica* isolate UMB-019.99* | 99 | GQ411316 | 0.8 | 10.9 | <0.1 | <0.1 |
| *Filosporella cf. annelidica* CCM F-11702* | 97 | KC834044 | 0.5 | 0.1 | 0.6 | 0.6 |
| *Articulospora tetracladia* strain CCM F-10113* | 94 | KP234360 | 0.2 | 0.0 | 0.6 | 0.7 |
| Uncultured fungus clone MOTU_3160_GOKCVWY06GWYX5 | 99 | JN905693 | 3.7 | 8.5 | <0.1 | <0.1 |
| *Flagellospora penicillioides* isolate UMB-304.05* | 99 | GQ411325 | 1.5 | 4.5 |  |  |
| *Flagellospora curvula* strain CB-M13* | 97 | KC834045 | 0.2 | <0.1 | 0.4 | 0.4 |
| *Articulospora tetracladia* strain CCM F-10113* | 95 | KP234360 | <0.1 | <0.1 | 0.8 | 0.6 |
| *Leptosphaerulina chartarum* genomic DNA | 99 | LK936369 | 4.0 | 2.6 | <0.1 | 0.2 |
| *Vibrissea filisporia* 18S ribosomal RNA gene | 97 | JX415338 | 0.5 | 0.1 | 0.8 | 0.8 |
| *Dimorphospora foliicola* isolate UMB-031.01* | 99 | GQ411311 | 6.1 | 0.8 | 0.2 | <0.1 |
| *Articulospora tetracladia* strain CCM F-10113* | 96 | KP234360 |  | <0.1 | 0.1 | 0.4 |
| Uncultured fungus clone 035A14187 | 97 | JX368617 | 1.0 | 1.2 | 0.1 | 0.1 |
| *Paraphaeosphaeria michotii* strain MFLUCC-13-0349 | 100 | KJ939279 | 4.7 | 0.3 | <0.1 | <0.1 |
| Uncultured fungus clone SG061_D07 | 96 | KP889636 |  | <0.1 | 0.4 | 0.1 |
| *Articulospora tetracladia* strain CCM F-10113* | 96 | KP234360 | 1.1 | 1.3 |  |  |
| Uncultured Erysiphaceae | 99 | DQ066421 |  |  | 0.7 |  |
| *Articulospora tetracladia* strain CCM F-10113* | 95 | KP234360 | 0.6 | 1.8 |  |  |
| *Flagellospora saccata* strain CCM F-39994* | 93 | KC834053 |  | <0.1 | 0.4 | <0.1 |
| *Articulospora tetracladia* strain CCM F-10113* | 97 | KP234360 | 0.1 | 0.3 | 0.1 | <0.1 |
| Uncultured fungus clone MOTU_3195_GYUGVSB04JY6XO | 97 | JN905720 | 2.3 | <0.1 |  | <0.1 |
| *Articulospora proliferata* strain CCM F-11200* | 92 | KP234351 | 1.2 | 0.6 |  | <0.1 |
| Uncultured Leotiomycetes clone FON_16e02 | 94 | HM488478 | <0.1 | 1.3 |  | <0.1 |
| Uncultured fungus clone Contig592-105-1140_1234 | 85 | FJ776609 |  | <0.1 | 0.2 | 0.2 |
| *Rachicladosporium cboliae* strain CPC 14034 | 98 | GU214650 | 1.8 | 0.3 |  | <0.1 |
| *Articulospora atra* strain CCM F-01384* | 99 | KP234353 |  |  | 0.2 | <0.1 |
| Uncultured fungus clone Singleton_49-3019_1447 | 95 | FJ761070 | 2.1 | 0.1 |  |  |
| *Neonectria* sp. I174 | 99 | GU062249 | 1.2 | 0.9 |  | <0.1 |
| Uncultured soil fungus clone BD48 | 100 | JQ666671 |  | 4.0 |  |  |
| Uncultured *Fusarium* clone r13 | 99 | KR063517 |  | <0.1 |  | 0.2 |
| Uncultured fungus clone MOTU_2920_GOKCVWY06G0ANO | 97 | JN905521 |  |  | 0.1 | 0.2 |
| Uncultured Lecanorales clone LTSP_EUKA_P1J14 | 91 | FJ552880 | 0.1 | <0.1 | 0.1 | <0.1 |
| *Tricladium obesum* strain CCM F-14598* | 97 | KC834068 | <0.1 | <0.1 | 0.4 | <0.1 |
| *Paraphoma radicina* isolate 36 | 99 | KP174683 |  |  | 0.5 | <0.1 |
| Uncultured fungus clone SG023_A04 | 97 | KP889774 | 1.9 | 0.3 |  |  |
| Uncultured fungus clone MOTU_3038_GYUGVSB04JBS12 | 91 | JN905603 | 0.5 | 2.1 |  |  |
| *Lophiostoma fuckelii* culture-collection CBS:113432 | 98 | EU552139 |  | 0.6 | <0.1 | <0.1 |
| *Glomus* sp. FO128-131 | 99 | FM253381 | 2.4 |  |  | <0.1 |
| *Teratosphaeria molleriana* strain CBS 118359 | 99 | EU167583 |  | 1.8 |  | <0.1 |
| *Microdochium bolleyi* isolate 20_45D | 99 | KC989068 |  |  | 0.1 | 0.1 |
| *Neofabraea alba* strain ID04 | 96 | KJ396077 |  | <0.1 | 0.1 | <0.1 |
| *Mortierella elongata* isolate F15 | 99 | JF439485 |  |  | 0.3 | <0.1 |
| *Podospora appendiculata* strain IFO 8549 | 99 | AY999126 |  |  | 0.3 | <0.1 |
| Uncultured fungus clone MOTU_2426_GOKCVWY06G4JNZ | 90 | JN905170 |  | 1.3 |  |  |
| *Tricladium obesum* strain CCM F-14598* | 97 | KC834068 | 0.2 | <0.1 | 0.2 | <0.1 |
| Uncultured Ascomycota isolate 790 | 98 | HM141062 |  | <0.1 | 0.1 | 0.1 |
| *Filosporella fistucella* strain CCM F-13091/ *Filosporella exilis* strain CCM F-13097* | 96 | KC834047 |  |  | 0.2 | <0.1 |
| Uncultured Discosia clone OS_2w_E01 | 99 | JF449727 | 1.1 | 0.3 |  |  |
| *Articulospora tetracladia* strain CCM F-10113* | 96 | KP234360 |  | <0.1 | <0.1 | <0.1 |
| Uncultured fungus clone OTU_50_468_11274 | 99 | KF222311 |  | 2.0 |  |  |
| Uncultured *Malassezia* clone IBL157f | 99 | KT334711 |  |  | 0.2 |  |
| *Alatospora acuminata* strain ccm-F13089* | 92 | AY204589 | 0.5 | <0.1 | <0.1 | <0.1 |
| *Dinemasporium strigosum* strain CBS 520.78 | 99 | JQ889282 |  | 0.6 |  |  |
| *Devriesia fraseriae* culture-collection CBS:128217 | 99 | HQ599602 |  | 1.1 |  |  |
| Uncultured fungus clone MOTU_3195_GYUGVSB04JY6XO | 98 | JN905720 | 0.3 | <0.1 |  | <0.1 |
| Uncultured Auriculariales clone OTU_99 | 98 | KT728279 | <0.1 | 0.3 |  |  |
| Uncultured fungus clone MOTU_1840_GOKCVWY06G673C | 98 | JN904763 | <0.1 | 0.2 | <0.1 | <0.1 |
| *Articulospora tetracladia* strain CBS 106.46* | 98 | KP234384 | 0.2 | <0.1 |  |  |
| Uncultured fungus clone U_QM_090722_258_Bb03.b1 | 98 | JN396401 |  |  | 0.1 | <0.1 |
| *Mycosphaerella mexicana* isolate AMR216/*Teratosphaeria mexicana* | 99 | AY509769 |  | 0.4 |  |  |
| Uncultured Ascomycota clone Mesq_D11 | 94 | EU490133 | 0.5 | 0.4 |  |  |
| *Flagellospora saccata* strain CCM F-39994* | 97 | KC834053 |  |  | <0.1 | <0.1 |
| Uncultured fungus clone Singleton_43-2968_3314 | 99 | FJ762796 | <0.1 | <0.1 | <0.1 | <0.1 |
| *Seimatosporium pseudorosarum* clone MFLUCC 14-0466 | 98 | KT284775 | 0.7 | <0.1 |  | <0.1 |
| *Articulospora tetracladia* strain CCM F-10113* | 94 | KP234360 | <0.1 | 0.3 |  |  |
| *Penidiella ellipsoidea* strain CBS 128773 | 99 | JF499843 |  | 0.3 |  |  |
| *Alatospora pulchella* strain CCM F-502* | 98 | KC834039 | 1.3 | 0.1 |  |  |
| Uncultured fungus clone CT129 | 97 | GU461445 | 0.5 | 0.9 |  |  |
| *Articulospora tetracladia* strain CCM F-10113* | 96 | KP234360 | <0.1 | 0.2 |  |  |
| *Articulospora tetracladia* strain CCM F-10113* | 95 | KP234360 | <0.1 | <0.1 | <0.1 | <0.1 |
| *Alatospora pulchella* strain CCM F-502* | 97 | KC834039 | 1.4 | <0.1 |  |  |
| *Articulospora tetracladia* strain CCM F-10113* | 96 | KP234360 | 0.3 | 0.1 |  |  |
| *Teratosphaeria parva* strain CBS 122894 | 99 | EU707877 |  | 0.2 |  | <0.1 |
| Uncultured fungus clone SG023_A04 | 95 | KP889774 | 1.5 | <0.1 |  |  |
| *Articulospora tetracladia* strain CCM F-10113* | 95 | KP234360 | <0.1 | 0.4 |  |  |
| *Alatospora pulchella* strain CCM F-502* | 92 | KC834039 |  | <0.1 | <0.1 | <0.1 |
| *Microdochium phragmitis* CBS 285.71 | 99 | AJ279449 | 0.2 | 0.1 |  |  |
| Uncultured fungus clone U_QM_090722_268_Af06.b1 | 99 | JN397343 | 0.3 | 0.1 |  |  |
| *Alnicola umbrina* strain TU110266 | 99 | JN943985 | 0.6 | <0.1 |  |  |
| *Epicoccum nigrum* strain G361 | 99 | KR094452 | <0.1 | <0.1 | <0.1 | <0.1 |
| *Lunulospora curvula* isolate UMB-499.09* | 99 | JX089527 |  | 0.1 |  |  |
| *Venturia hystrioides* strain CBS 117727 | 99 | EU035459 | <0.1 | <0.1 |  | <0.1 |
| *Articulospora tetracladia* strain CCM F-10113* | 98 | KP234360 | <0.1 | <0.1 | <0.1 | <0.1 |
| *Cladosporium nigrellum* strain ATCC 200937 | 98 | AF393719 |  | <0.1 |  | <0.1 |
| Uncultured fungus clone MOTU_284_GYUGVSB04IYRCZ | 85 | JN905465 | <0.1 | <0.1 | <0.1 | <0.1 |
| *Thelonectria discophora* culture-collection CBS:125487 | 98 | HQ897789 | <0.1 | <0.1 | <0.1 | <0.1 |
| *Articulospora tetracladia* strain CCM F-14298* | 96 | EU998923 |  | <0.1 | <0.1 | <0.1 |
| Uncultured fungus clone MOTU_1840_GOKCVWY06G673C | 97 | JN904763 |  | 0.2 |  |  |
| Ascomycete sp. olrim401 | 93 | AY781244 | <0.1 | 0.2 |  |  |
| *Filosporella cf. annelidica* CCM F-11702* | 97 | KC834044 | <0.1 | 0.2 | <0.1 | <0.1 |
| Uncultured Erysiphaceae | 99 | DQ066421 |  | <0.1 | <0.1 |  |
| Uncultured Mortierella clone LTSP_EUKA_P4O11 | 99 | FJ553914 |  |  | 0.1 | <0.1 |
| *Flagellospora saccata* strain CCM F-39994* | 89 | KC834053 | <0.1 | <0.1 |  |  |
| *Articulospora tetracladia* strain CCM F-10113* | 89 | KP234360 | <0.1 | 0.1 |  |  |
| Uncultured fungus clone SW008_H12 | 96 | KP889776 |  |  | <0.1 | <0.1 |
| *Catenulostroma hermanusense* strain CBS 128768 | 96 | JF499833 |  | 0.5 |  |  |
| *Articulospora proliferata* strain CCM F-11200* | 96 | KP234351 | 0.2 | <0.1 |  |  |
| *Articulospora tetracladia* strain CCM F-10113* | 96 | KP234360 |  |  | <0.1 | <0.1 |
| *Fontanospora fusiramosa* strain CCM F-11313* | 95 | KP234355 |  |  | <0.1 | <0.1 |
| *Alatospora acuminata* strain ccm-F13089* | 95 | AY204589 |  |  | 0.0 |  |
| *Articulospora tetracladia* strain CCM F-10113* | 97 | KP234360 |  | <0.1 | <0.1 | <0.1 |
| *Botrytis cinerea* B05.10 | 100 | CP009808 |  | 0.3 |  | <0.1 |
| *Montagnula scabiosae* strain MFLUCC 14-0954 | 99 | KT443907 |  | 0.2 |  |  |
| *Paraconiothyrium brasiliense* isolate F01 | 99 | JF439492 | <0.1 | <0.1 | <0.1 | <0.1 |
| *Fontanospora fusiramosa* strain CCM F-11313* | 96 | KP234355 |  |  | <0.1 | <0.1 |
| Uncultured fungus clone Unisequence#69-3354_2750 | 99 | GQ524076 |  | <0.1 |  | <0.1 |
| *Articulospora tetracladia* strain CCM F-14298* | 90 | EU998923 |  |  |  | <0.1 |
| *Phoma herbarum* genomic DNA | 98 | FN868459 |  | <0.1 |  | <0.1 |
| Ascomycete sp. olrim401 | 94 | AY781244 |  |  | <0.1 | <0.1 |
| Uncultured fungus clone CMH115 | 96 | KF800206 |  |  | <0.1 | <0.1 |
| *Varicosporium elodeae* strain WA0000019152* | 97 | JX981463 |  |  | <0.1 | <0.1 |
| *Vibrissea filisporia* 18S ribosomal RNA gene | 95 | JX415338 |  |  | <0.1 | <0.1 |
| *Glomerella acutata* strain JF17 | 99 | FJ455523 |  | <0.1 |  |  |
| *Epicoccum nigrum* 18S ribosomal RNA gene | 99 | FJ904918 | <0.1 | <0.1 | <0.1 | <0.1 |
| *Flagellospora saccata* strain CCM F-39994* | 94 | KC834053 |  |  | <0.1 | <0.1 |
| *Articulospora tetracladia* strain CCM F-10113* | 95 | KP234360 | <0.1 | 0.1 |  |  |
| Uncultured ectomycorrhizal fungus genes | 98 | AB669512 |  |  |  | <0.1 |
| Uncultured fungus clone MOTU_437_G2Q8WDE03C34U2 | 99 | JN906555 |  | 0.4 |  |  |
| Uncultured fungus clone MOTU_51_GOKCVWY06GU9GB | 99 | JN906617 | <0.1 | <0.1 |  | <0.1 |
| *Nectria diminuta* culture-collection CBS:114636 | 99 | HQ897813 |  | <0.1 |  | <0.1 |
| *Alatospora pulchella* strain CCM F-502* | 87 | KC834039 |  |  | <0.1 | <0.1 |
| Uncultured Sordariomycetes clone AhedenH7 | 88 | FJ475695 |  |  | <0.1 | <0.1 |
| *Flagellospora curvula* strain CB-M13* | 95 | KC834045 |  |  |  | <0.1 |
| *Articulospora tetracladia* strain CCM F-10113* | 99 | KP234360 | <0.1 | <0.1 | <0.1 | <0.1 |
| Uncultured fungus clone CMH112 | 99 | KF800203 |  | <0.1 |  | <0.1 |
| *Articulospora tetracladia* strain CCM F-10113* | 93 | KP234360 |  | <0.1 |  | <0.1 |
| Uncultured fungus clone OTU#2354-63-3414_0957 | 99 | GQ510604 |  | 0.1 |  |  |
| Uncultured fungus clone OTU#2950-61-3429_1608 | 95 | GQ511198 |  |  |  | <0.1 |
| *Thelonectria discophora* culture-collection CBS:125487 | 95 | HQ897789 |  |  | <0.1 | <0.1 |
| *Flagellospora curvula* strain CB-M13* | 95 | KC834045 |  |  |  | <0.1 |
| *Flagellospora curvula* strain CB-M13* | 94 | KC834045 |  |  | <0.1 | <0.1 |
| *Filosporella cf. annelidica* CCM F-11702* | 95 | KC834044 |  |  | <0.1 | <0.1 |
| *Gorgomyces honrubiae* strain CCM F-12003* | 93 | KC834057 |  |  | <0.1 | <0.1 |
| *Hymenoscyphus monotropae* voucher CC 19-47 | 96 | KF359569 |  | <0.1 | <0.1 | <0.1 |
| Uncultured fungus clone MOTU_1840_GOKCVWY06G673C | 98 | JN904763 | 0.1 | <0.1 |  |  |
| *Articulospora tetracladia* strain CCM F-10113* | 97 | KP234360 |  | <0.1 |  | <0.1 |
| *Articulospora tetracladia* strain CCM F-10113* | 95 | KP234360 | <0.1 | <0.1 |  |  |
| *Varicosporium elodeae* strain WA0000019152* | 93 | JX981463 |  |  | <0.1 | <0.1 |
| *Vibrissea filisporia* 18S ribosomal RNA gene | 91 | JX415338 |  |  | <0.1 | <0.1 |
| Ascomycete sp. olrim401 | 94 | AY781244 |  |  | <0.1 | <0.1 |
| *Articulospora tetracladia* strain CCM F-10113* | 95 | KP234360 | <0.1 | <0.1 | <0.1 | <0.1 |
| *Articulospora tetracladia* strain CCM F-10113* | 91 | KP234360 |  |  | <0.1 | <0.1 |
| *Flagellospora curvula* strain CB-M13* | 92 | KC834045 |  |  | <0.1 | <0.1 |
| *Articulospora tetracladia* strain CCM F-10113* | 97 | KP234360 |  |  | <0.1 | <0.1 |
| *Monochaetia kansensis* strain ZJLQ463 | 99 | KC345691 |  |  |  | <0.1 |
| Uncultured Erysiphaceae | 97 | DQ066421 |  | 0.1 | <0.1 |  |
| *Phoma herbarum* strain ASR_H56_24A | 99 | JX421725 |  | <0.1 |  | <0.1 |
| Uncultured fungus clone 035A11278 | 98 | JX365757 |  | <0.1 |  |  |
| Uncultured fungus clone U_QM_090722_76_Ae03.b1 | 99 | JN397005 | 0.1 | <0.1 |  |  |
| *Articulospora atra* strain CCM F-01384* | 98 | KP234353 |  |  | <0.1 | <0.1 |
| *Capnobotryella* sp. MA 4642 | 98 | AJ971406 |  |  |  | <0.1 |
| Uncultured fungus clone MOTU_3160_GOKCVWY06GWYX5 | 96 | JN905693 |  | 0.2 |  |  |
| *Varicosporium elodeae* strain WA0000019152* | 97 | JX981463 |  | <0.1 |  | <0.1 |
| *Antennariella placitae* ITS region | 99 | NR_132831 |  | <0.1 |  |  |
| *Flagellospora saccata* strain CCM F-39994* | 87 | KC834053 |  | <0.1 |  |  |
| *Catenulostroma hermanusense* strain CBS 128768 | 99 | JF499833 |  | <0.1 |  |  |
| *Clavariopsis aquatica*18S ribosomal RNA gene* | 96 | GQ152143 | <0.1 | <0.1 |  |  |
| *Flagellospora saccata* strain CCM F-39994* | 96 | KC834053 |  |  |  | <0.1 |
| *Monodictys arctica* strain UAMH 10719 | 97 | EU686520 | <0.1 | <0.1 |  |  |
| Uncultured fungus clone 106_NA11_P33_A24 | 97 | KC965976 |  |  | <0.1 | <0.1 |
| *Articulospora tetracladia* strain CCM F-14298* | 95 | EU998923 |  |  | <0.1 | <0.1 |
| Uncultured fungus clone MOTU_2960_GOKCVWY06G12WS | 99 | JN905545 |  | <0.1 |  |  |
| Uncultured fungus clone SG061_D07 | 92 | KP889636 |  |  |  | <0.1 |
| *Articulospora tetracladia* strain CBS 106.46* | 93 | KP234384 |  | <0.1 |  | <0.1 |
| Uncultured fungus clone MOTU_2243_GOKCVWY06GWIQ4 | 99 | JN905046 |  |  |  | <0.1 |
| *Articulospora tetracladia* strain CCM F-14299* | 93 | KP234360 |  | <0.1 |  | <0.1 |
| *Catenulostroma hermanusense* strain CBS 128768 | 96 | JF499833 |  | <0.1 |  |  |
| *Flagellospora curvula* strain CB-M13* | 93 | KC834045 |  |  |  | <0.1 |
| *Orbilia sp. 'rosea'* 18S ribosomal RNA gene | 97 | KM199779 |  |  |  | <0.1 |
| *Venturia atriseda* strain CBS 371.55 | 97 | EU035448 |  | <0.1 |  | <0.1 |
| *Articulospora tetracladia* strain CCM F-14299* | 94 | KP234360 |  |  | <0.1 | <0.1 |
| *Orbilia sp. 'rosea'* 18S ribosomal RNA gene | 93 | KM199779 |  |  |  | <0.1 |
| *Rachicladosporium cboliae* strain CPC 14034 | 97 | GU214650 | <0.1 | <0.1 |  |  |
| Uncultured fungus clone MOTU_2562_GOKCVWY06G38AF | 99 | JN905263 | <0.1 | <0.1 | <0.1 |  |
| Uncultured fungus clone MOTU_2591_GOKCVWY06GSTXH | 93 | JN905286 |  |  | <0.1 | <0.1 |
| *Articulospora tetracladia* strain CCM F-14299* | 98 | KP234360 | <0.1 | <0.1 | <0.1 | <0.1 |
| *Lunulospora curvula* isolate UMB-499.09* | 97 | JX089527 | <0.1 | <0.1 |  |  |
| *Tricladium obesum* strain CCM F-14598* | 97 | KC834068 | <0.1 | <0.1 |  |  |
| Uncultured fungus clone MOTU_862_GYUGVSB04IYKEH | 98 | JN906872 |  |  |  | <0.1 |
| *Articulospora tetracladia* strain CCM F-10113* | 91 | KP234360 | <0.1 | <0.1 |  |  |
| *Paraconiothyrium archidendri* strain CBS 168.77 | 99 | JX496049 |  | <0.1 |  |  |
| Uncultured fungus clone MOTU_1431_GOKCVWY06HB3UZ | 99 | JN904461 |  |  |  | <0.1 |
| *Alternaria brassicae* 18S ribosomal RNA gene | 97 | AY154714 |  | <0.1 |  |  |
| *Articulospora proliferata* strain CCM F-11200* | 91 | KP234351 | <0.1 | <0.1 |  |  |
| *Articulospora tetracladia* strain CCM F-10113* | 94 | KP234360 |  |  | <0.1 | <0.1 |
| *Alatospora pulchella* strain CCM F-502* | 93 | KC834039 |  | <0.1 |  |  |
| *Articulospora proliferata* strain CCM F-11200* | 92 | KP234351 |  | <0.1 |  |  |
| *Articulospora tetracladia* strain CCM F-10113* | 95 | KP234360 | <0.1 | <0.1 |  |  |
| *Cladosporium cladosporioides* 18S rRNA gene | 99 | AJ300335 |  | <0.1 |  |  |
| *Cryptococcus* sp. 6-13 | 99 | KT372800 |  | <0.1 |  |  |
| *Penidiella ellipsoidea* strain CBS 128773 | 96 | JF499843 |  | <0.1 |  |  |
| *Sclerostagonospora* sp. CBS 118152 | 99 | JX517283 |  | <0.1 |  |  |
| Uncultured fungus clone MOTU_1421_GOKCVWY06HD8P9 | 99 | JN904456 |  |  |  | <0.1 |
| Uncultured soil fungus clone 137-18 | 96 | DQ421226 |  | <0.1 |  |  |
| *Varicosporium elodeae* strain WA0000019152* | 95 | JX981463 |  |  |  | <0.1 |
| *Articulospora tetracladia* strain CCM F-10113* | 94 | KP234360 | <0.1 | <0.1 |  |  |
| *Flagellospora curvula* strain CB-M13* | 96 | KC834045 |  | <0.1 |  | <0.1 |
| *Leptosphaerulina* sp. DBCMVB | 98 | KJ867213 | <0.1 | <0.1 |  |  |
| *Tyromyces chioneus* voucher UBCF23782 | 98 | KC581305 |  |  | <0.1 |  |
| Uncultured fungus clone MOTU_614_GYUGVSB04IPO8J | 95 | JN906687 |  | <0.1 |  |  |
| Uncultured fungus clone SG023_A04 | 95 | KP889774 | 0.1 | <0.1 |  |  |
| Uncultured Lecanorales clone LTSP_EUKA_P1J14 | 91 | FJ552880 |  |  | <0.1 | <0.1 |
| *Articulospora tetracladia* strain CBS 106.46* | 97 | KP234384 | <0.1 | <0.1 |  | <0.1 |
| *Articulospora tetracladia* strain CCM F-03680c* | 92 | EU998926 |  | <0.1 |  | <0.1 |
| *Articulospora tetracladia* strain CCM F-10113* | 96 | KP234360 |  | <0.1 |  |  |
| *Articulospora tetracladia* strain CCM F-10113* | 96 | KP234360 |  |  |  | <0.1 |
| *Claviceps paspali* genes for 18S rRNA | 98 | AB250409 |  | <0.1 |  |  |
| *Neoerysiphe galeopsidis* specimen_voucher: KW:33697F, MUMH 4657 | 99 | AB498940 |  | <0.1 |  |  |
| *Sclerostagonospora cycadis* strain CBS 291.76 | 99 | KR611890 | <0.1 | <0.1 |  | <0.1 |
| Uncultured fungus clone HS3-31 | 98 | KU141315 | <0.1 | <0.1 |  |  |
| Uncultured fungus clone N3D28_27 | 92 | HQ445274 |  | <0.1 |  |  |
| Uncultured soil fungus clone BL21 | 99 | JQ666340 |  | <0.1 |  |  |
| *Flagellospora fusarioides* strain CCM F-14583* | 91 | KC834048 |  |  |  | <0.1 |
| *Fontanospora fusiramosa* strain CCM F-11313* | 94 | KP234355 | <0.1 | <0.1 |  |  |
| *Nigrospora sphaerica* genomic DNA | 100 | LN809021 |  |  |  | <0.1 |
| *Teratosphaeria pseudafricana* strain PM16 | 98 | JN232423 |  |  |  | <0.1 |
| Uncultured fungus clone 036A23583 | 95 | JX377779 |  | <0.1 |  |  |
| Uncultured fungus clone LMRF_66 | 99 | GU078632 |  | <0.1 |  | <0.1 |
| Uncultured fungus clone OTU_951_GW5CJXV07IMV3E | 97 | JF945083 |  | <0.1 |  | <0.1 |
| *Flagellospora curvula* strain CB-M13* | 92 | KC834045 |  | <0.1 |  | <0.1 |
| Uncultured ectomycorrhiza (Leotiomycetes) clone LTSP_EUKA_P6B23 | 96 | FJ554268 |  |  |  | <0.1 |
| Uncultured fungus clone MOTU_1973_GOKCVWY06GZJIU | 99 | JN904860 |  | <0.1 |  |  |
| Uncultured fungus clone MOTU_2252_GYUGVSB04H83BQ | 99 | JN905054 |  | <0.1 |  | <0.1 |
| Uncultured fungus clone MOTU_3253_GOKCVWY06G3NZ9 | 96 | JN905762 |  |  |  | <0.1 |
| *Cylindrocarpon pauciseptatum* strain Cy238 | 100 | JF735307 |  | <0.1 |  |  |
| *Flagellospora curvula* strain CB-M13* | 95 | KC834045 |  |  |  | <0.1 |
| *Flagellospora saccata* strain CCM F-39994* | 89 | KC834053 |  | <0.1 |  |  |
| Fungal endophyte sp. ICMP 16120 | 93 | EU482201 | <0.1 | <0.1 |  |  |
| *Ilyonectria liriodendri* strain Cy122 | 97 | JF735261 |  | <0.1 |  |  |
| *Lophiostoma corticola* strain Fst64 | 98 | KC464348 |  | <0.1 |  | <0.1 |
| *Lophiostoma fuckelii* culture-collection CBS:113432 | 98 | EU552139 |  | <0.1 |  |  |
| *Penicillium spinulosum* small subunit ribosomal RNA gene | 100 | KF646101 |  | <0.1 |  | <0.1 |
| *Tricladium chaetocladium* strain UMB-854.11* | 99 | KF952683 |  | <0.1 |  |  |
| Uncultured fungus clone MOTU_3639_GOKCVWY06HEW3C | 96 | JN906053 |  | <0.1 |  |  |
| Uncultured soil fungus clone A20 | 99 | HM037656 |  | <0.1 |  |  |
| *Articulospora tetracladia* strain CCM F-10113* | 95 | KP234360 |  | <0.1 |  |  |
| *Articulospora tetracladia* strain CCM F-10113* | 97 | KP234360 |  |  |  | <0.1 |
| *Articulospora tetracladia* strain CCM F-11805* | 89 | EU998928 |  |  | <0.1 | <0.1 |
| *Articulospora tetracladia* strain CCM F-11805* | 93 | EU998928 |  |  |  | <0.1 |
| Ascomycota sp. I364 | 99 | GU062306 |  |  | <0.1 | <0.1 |
| Ascomycota sp. UNEX FECRGA 2012E266 | 100 | KP899437 |  | <0.1 |  |  |
| *Colletotrichum acutatum* 18S rRNA gene | 99 | AJ301971 |  |  |  | <0.1 |
| *Lunulospora curvula* isolate UMB-499.09* | 98 | JX089527 |  | <0.1 |  |  |
| *Periconia* sp. 0511ARD16P1 | 95 | LN808857 |  | <0.1 |  | <0.1 |
| Uncultured fungus clone 035A13809 | 99 | JX368249 |  |  |  | <0.1 |
| Uncultured fungus clone OTU_1689_77_5532 | 99 | KF221505 |  | <0.1 |  |  |
| Uncultured fungus clone Unisequence#61-3282_2843 | 93 | GQ526591 |  | <0.1 |  |  |
| Uncultured Lecanorales clone LTSP_EUKA_P1J14 | 92 | FJ552880 |  |  |  | <0.1 |
| *Varicosporium elodeae* strain WA0000019152* | 97 | JX981463 |  |  | <0.1 | <0.1 |
| *Alatospora flagellata* strain CCM F-501* | 95 | KC834041 |  |  |  | <0.1 |
| *Articulospora tetracladia* strain CBS 106.46* | 97 | KP234384 |  | <0.1 |  |  |
| *Articulospora tetracladia* strain CCM F-10113* | 94 | KP234360 |  |  |  | <0.1 |
| *Articulospora tetracladia* strain CCM F-10113* | 93 | KP234360 |  |  |  | <0.1 |
| *Cladosporium rhusicola* culture-collection CPC:15219 | 100 | KT600440 | <0.1 | <0.1 |  |  |
| *Didymella rosea* voucher TAS 042-0012 | 99 | KT287020 |  |  |  | <0.1 |
| *Discosia pseudoartocreas* CBS 136438 | 93 | NR_132068 |  | <0.1 |  | <0.1 |
| *Flagellospora curvula* strain CB-M13* | 94 | KC834045 |  |  |  | <0.1 |
| *Flagellospora saccata* strain CCM F-39994* | 95 | KC834053 |  |  |  | <0.1 |
| *Flagellospora saccata* strain CCM F-39994* | 94 | KC834053 |  |  |  | <0.1 |
| Fungal sp. TRN499 | 99 | AY843181 |  | <0.1 |  |  |
| *Fusicladium mandshuricum* strain CBS 112235 | 96 | EU035433 |  | <0.1 |  | <0.1 |
| *Paraphaeosphaeria sporulosa* strain CBS 401.71 | 95 | JX496084 |  |  |  | <0.1 |
| *Trichocladium asperum* isolate PRF02 | 99 | KC311502 |  | <0.1 |  |  |
| Uncultured fungus genes | 88 | AB580909 | <0.1 |  |  |  |
| *Verticillium* sp. I88 | 94 | GU062214 |  | <0.1 |  |  |
| *Xenopolyscytalum pinea* culture-collection CPC:14225 | 99 | HQ599580 |  |  |  | <0.1 |
| *Alatospora pulchella* strain CCM F-502* | 87 | KC834039 |  | <0.1 |  |  |
| *Bisporella citrina* voucher ILLS:61033 | 93 | JQ256414 |  | <0.1 |  |  |
| *Flagellospora curvula* strain CB-M13* | 95 | KC834045 |  |  |  | <0.1 |
| Fungal sp. ARIZ B179cA | 81 | FJ612951 | <0.1 |  |  |  |
| *Fusarium cortaderiae* isolate EFA 6FCMG | 99 | KF576625 |  | <0.1 |  |  |
| *Neofabraea alba* strain ID04 | 95 | KJ396077 |  | <0.1 |  | <0.1 |
| *Paraphaeosphaeria michotii* strain CBS 340.86 | 98 | JX496079 | <0.1 |  | <0.1 |  |
| *Peziza badia* voucher 14638 | 97 | JF908544 |  | <0.1 |  |  |
| *Phoma exigua var. exigua* clone 26its22 | 99 | EU343168 |  | <0.1 |  | <0.1 |
| *Spirosphaera floriformis* strain CBS 402.52 | 99 | HQ696658 |  |  |  | <0.1 |
| *Tricladium minutum* 18S ribosomal RNA gene* | 93 | JQ412863 |  |  |  | <0.1 |
| Uncultured Ascomycota clone LTSP_EUKA_P5B18 | 86 | FJ553969 |  |  |  | <0.1 |
| Uncultured fungus clone MOTU_1647_GOKCVWY06HA8CT | 99 | JN904620 |  | <0.1 |  |  |
| *Variocladium giganteum* CBS 508.71* | 96 | NR_111206 |  |  | <0.1 | <0.1 |
| *Alatospora flagellata* strain CCM F-501* | 97 | KC834041 |  |  | <0.1 | <0.1 |
| *Articulospora tetracladia* strain CCM F-10113* | 95 | KP234360 |  | <0.1 |  |  |
| *Articulospora tetracladia* strain CCM F-10113* | 95 | KP234360 |  | <0.1 |  |  |
| *Articulospora tetracladia* strain CCM F-10113* | 98 | KP234360 |  | <0.1 |  |  |
| *Articulospora tetracladia* strain CCM F-10113* | 87 | KP234360 |  |  |  | <0.1 |
| *Articulospora tetracladia* strain CCM F-11805* | 92 | EU998928 |  |  | <0.1 | <0.1 |
| *Bisporella citrina* voucher ILLS:61033 | 93 | JQ256414 |  |  |  | <0.1 |
| *Deconica citrispora* voucher PDD:87522 | 98 | KM975431 |  | <0.1 |  |  |
| *Filosporella cf. annelidica* CCM F-11702* | 99 | KC834044 |  |  | <0.1 |  |
| *Flagellospora curvula* strain CB-M13* | 95 | KC834045 |  |  |  | <0.1 |
| *Flagellospora curvula* strain CB-M13* | 93 | KC834045 |  |  |  | <0.1 |
| *Flagellospora saccata* strain CCM F-39994* | 89 | KC834053 |  | <0.1 |  |  |
| *Fontanospora fusiramosa* strain CCM F-11313* | 95 | KP234355 |  |  |  | <0.1 |
| *Neophysalospora eucalypti* culture-collection CBS:138864 | 99 | KP004462 |  | <0.1 |  |  |
| *Stylonectria applanata* culture-collection CBS:125489 | 100 | HQ897805 |  |  |  | <0.1 |
| *Teratosphaeriopsis pseudafricana* strain CBS 111171 | 97 | KF901738 |  | <0.1 |  |  |
| Uncultured ascomycete ITS region | 99 | AM901813 |  | <0.1 |  |  |
| Uncultured Ascomycota clone 430 | 92 | HM239990 |  |  |  | <0.1 |
| Uncultured Erysiphaceae | 99 | DQ066421 | <0.1 |  |  |  |
| Uncultured fungus clone 038A40009 | 97 | JX356858 |  | <0.1 |  |  |
| Uncultured fungus clone 106_NA11_P33_A24 | 92 | KC965976 |  |  |  | <0.1 |
| Uncultured fungus clone 107A68083 | 91 | JX334160 |  |  |  | <0.1 |
| Uncultured fungus clone 3270A8 | 97 | KF617316 |  |  |  | <0.1 |
| Uncultured fungus clone MOTU_3930_GYUGVSB04JW4D2 | 87 | JN906262 |  | <0.1 |  |  |
| Uncultured fungus clone N145 | 89 | JF300564 |  |  |  | <0.1 |
| Uncultured fungus clone OTU_361_GW5CJXV07IPNX5 | 99 | JF944982 |  | <0.1 |  | <0.1 |
| Uncultured fungus clone SG023_A04 | 96 | KP889774 | <0.1 | <0.1 |  |  |
| Uncultured fungus clone Singleton_28-2763_3239 | 97 | FJ760606 | <0.1 |  |  |  |
| Uncultured *Sterigmatomyces* clone Leof66 | 80 | KF225855 |  |  |  | <0.1 |
| *Varicosporium elodeae* strain WA0000019152* | 97 | JX981463 |  |  |  | <0.1 |
| *Alatospora acuminata* strain ccm-F13089* | 89 | AY204589 | <0.1 | <0.1 |  |  |
| *Alatospora flagellata* strain CCM F-501* | 97 | KC834041 |  |  | <0.1 |  |
| *Alatospora pulchella* strain CCM F-502* | 88 | KC834039 |  |  |  | <0.1 |
| *Anguillospora filiformis* isolate UMB-704.11* | 99 | JX089461 |  | <0.1 |  |  |
| *Articulospora atra* strain CCM F-01384* | 88 | KP234353 |  | <0.1 |  |  |
| *Articulospora tetracladia* strain CBS 106.46* | 97 | KP234384 |  | <0.1 |  |  |
| *Articulospora tetracladia* strain CCM F-10113* | 96 | KP234360 |  |  | <0.1 | <0.1 |
| *Articulospora tetracladia* strain CCM F-10113* | 95 | KP234360 |  |  | <0.1 |  |
| *Articulospora tetracladia* strain CCM F-10113* | 93 | KP234360 |  | <0.1 |  |  |
| *Articulospora tetracladia* strain CCM F-10113* | 94 | KP234360 |  |  |  | <0.1 |
| *Articulospora tetracladia* strain CCM F-10113* | 97 | KP234360 |  |  |  | <0.1 |
| *Articulospora tetracladia* strain CCM F-11805* | 92 | EU998928 |  |  |  | <0.1 |
| *Articulospora tetracladia* strain CCM F-14298* | 96 | EU998923 |  |  |  | <0.1 |
| *Articulospora tetracladia* strain CCM F-14298* | 96 | EU998923 |  | <0.1 |  | <0.1 |
| *Dendryphion europaeum* strain CPC 22943 | 100 | KJ869146 |  | <0.1 |  |  |
| *Elaphocordyceps inegoensis* genes | 99 | AB027368 |  |  |  | <0.1 |
| *Embellisia eureka* EGS 36-103 | 98 | AF392989 |  |  |  | <0.1 |
| *Filosporella versimorpha* strain CCM F-11194* | 94 | KC834054 |  | <0.1 |  |  |
| *Flagellospora curvula* strain CB-M13* | 93 | KC834045 |  |  | <0.1 | <0.1 |
| *Flagellospora curvula* strain CB-M13* | 98 | KC834045 |  |  | <0.1 | <0.1 |
| *Flagellospora saccata* strain CCM F-39994* | 91 | KC834053 |  |  | <0.1 | <0.1 |
| *Fontanospora fusiramosa* strain CCM F-11313* | 96 | KP234355 |  |  | <0.1 | <0.1 |
| Fungal sp. C22 AR-2014 | 98 | KF527820 |  | <0.1 |  |  |
| *Fusarium larvarum* strain BLD5 | 96 | FN868469 |  | <0.1 |  |  |
| *Hymenoscyphus monotropae* voucher CC 19-47 | 94 | KF359569 |  | <0.1 |  | <0.1 |
| *Infundichalara microchona* voucher CC 14-48 | 98 | KF359590 |  |  |  | <0.1 |
| *Mycosphaerella graminicola* strain CBS 100335 | 98 | EU019297 |  |  |  | <0.1 |
| *Neofabraea alba* strain ID04 | 93 | KJ396077 |  | <0.1 |  |  |
| *Penidiella ellipsoidea* strain CBS 128773 | 91 | JF499843 |  | <0.1 |  |  |
| *Pestalotiopsis* sp. 338p | 99 | KM507773 |  | <0.1 |  |  |
| Uncultured fungus clone 036A29166 | 98 | JX383191 |  |  |  | <0.1 |
| Uncultured fungus clone 109A77540 | 97 | JX389250 |  |  | <0.1 |  |
| Uncultured fungus clone 236967 | 89 | KP867316 |  | <0.1 |  |  |
| Uncultured fungus clone 38_NA4_P32_B6 | 81 | KC965223 |  |  | <0.1 |  |
| Uncultured fungus clone LT8 | 94 | HQ701748 |  | <0.1 |  |  |
| Uncultured fungus clone MOTU_10_GOKCVWY06G1DGV | 99 | JN904225 | <0.1 |  |  |  |
| Uncultured fungus clone MOTU_3298_GOKCVWY06GRT4E | 99 | JN905792 |  |  |  | <0.1 |
| Uncultured fungus clone MOTU_783_GYUGVSB04H4KL2 | 93 | JN906812 |  |  | <0.1 |  |
| Uncultured fungus clone OTU_1854_427_34960 | 99 | KF221550 |  | <0.1 |  |  |
| Uncultured fungus clone SG061_D07 | 95 | KP889636 |  |  | <0.1 |  |
| Uncultured fungus clone SW008_H12 | 95 | KP889776 |  |  |  | <0.1 |
| Uncultured fungus clone U_QM_090722_102_Be07.b1 | 91 | JN395462 |  |  | <0.1 | <0.1 |
| Uncultured Helotiales clone LTSP_EUKA_P1B19 | 99 | FJ552732 |  |  |  | <0.1 |
| Nº of OTUs |  |  | 109 | 232 | 120 | 219 |
