# Supplementary material for: Taxa-area relationship of aquatic fungi on deciduous leaves
Source: PLoS One. 2017 Jul 18;12(7):e0181545. doi: 10.1371/journal.pone.0181545 (PMC5515451; doi:10.1371/journal.pone.0181545)
Supplement: S4 Table — The exponential (EC) (Eq 4) and the logistic (LC) models (Eq 5) [4] were used. (DOCX) [file pone.0181545.s007.docx]

**S4 Table.** **Model parameters of the relationship between fungal taxa diversity and leaf area (Fig 4a, c) in TAR curves.** The exponential (EC) (equation 4) and the logistic (LC) models (equation 5) [4] were used.

|  | Stream | Model | Parameter | Parameter value | r^2^ | P |
| --- | --- | --- | --- | --- | --- | --- |
| Morphospecies | Oliveira Stream | EC | z | 0.97 | 0.21 | 0.4 |
|  |  |  | c | 18.25 |  |  |
|  | Boss Brook | EC | z | 3.29 | 0.88 | 0.005 |
|  |  |  | c | 15.67 |  |  |
|  | Oliveira Stream | LC | b | No fit | - | - |
|  |  |  | c | No fit |  |  |
|  |  |  | z | No fit |  |  |
|  | Boss Brook | LC | b | 39.66 | 0.92 | 0.0003 |
|  |  |  | c | 1.54 |  |  |
|  |  |  | z | 0.74 |  |  |
| OTUs | Oliveira Stream | EC | z | 22.44 | 0.90 | 0.004 |
|  |  |  | c | 89.48 |  |  |
|  | Boss Brook | EC | z | 14.50 | 0.59 | 0.08 |
|  |  |  | c | 108.0 |  |  |
|  | Oliveira Stream | LC | b | 232.8 | 0.97 | 0.0001 |
|  |  |  | c | 1.59 |  |  |
|  |  |  | z | 1.09 |  |  |
|  | Boss Brook | LC | b | 731.1 | 0.93 | 0.0002 |
|  |  |  | c | 5.33 |  |  |
|  |  |  | z | 2.32 |  |  |
