# Supplementary material for: Taxa-area relationship of aquatic fungi on deciduous leaves
Source: PLoS One. 2017 Jul 18;12(7):e0181545. doi: 10.1371/journal.pone.0181545 (PMC5515451; doi:10.1371/journal.pone.0181545)
Supplement: S5 Table — The exponential (EC) (Eq 4) and the logistic (LC) models (Eq 5) [4] were used. (DOCX) [file pone.0181545.s008.docx]

**S5 Table.** **Model parameters of the relationship between fungal taxa diversity and leaf area (Fig 4b, d) in TAC curves.** The exponential (EC) (equation 4) and the logistic (LC) models (equation 5) [4] were used.

|  | Stream | Model | Parameter | Parameter value | r^2^ | P |
| --- | --- | --- | --- | --- | --- | --- |
| Cumulative morphospecies | Oliveira Stream | EC | z | 3.40 | 0.90 | 0.004 |
|  |  |  | c | 19.72 |  |  |
|  | Boss Brook | EC | z | 5.00 | 0.92 | 0.002 |
|  |  |  | c | 19.26 |  |  |
|  | Oliveira Stream | LC | b | 71.90 | 0.98 | 0.00001 |
|  |  |  | c | 2.59 |  |  |
|  |  |  | z | 1.11 |  |  |
|  | Boss Brook | LC | b | 52.35 | 0.99 | 0.00003 |
|  |  |  | c | 1.68 |  |  |
|  |  |  | z | 1.20 |  |  |
| Cumulative OTUs | Oliveira Stream | EC | z | 51.40 | 0.99 | <0.0001 |
|  |  |  | c | 103.2 |  |  |
|  | Boss Brook | EC | z | 41.56 | 0.92 | 0.002 |
|  |  |  | c | 123.5 |  |  |
|  | Oliveira Stream | LC | b | 162.5 | 1.00 | 0.000002 |
|  |  |  | c | 0.61 |  |  |
|  |  |  | z | 0.91 |  |  |
|  | Boss Brook | LC | b | 283.7 | 1.00 | 0.000002 |
|  |  |  | c | 1.29 |  |  |
|  |  |  | z | 1.35 |  |  |
